# Oligodendrocyte–axon metabolic coupling is mediated by extracellular K^+^ and maintains axonal health

**DOI:** 10.1038/s41593-023-01558-3

**Published:** 2024-01-24

**Authors:** Zoe J. Looser, Zainab Faik, Luca Ravotto, Henri S. Zanker, Ramona B. Jung, Hauke B. Werner, Torben Ruhwedel, Wiebke Möbius, Dwight E. Bergles, L. Felipe Barros, Klaus-Armin Nave, Bruno Weber, Aiman S. Saab

**Affiliations:** 1 Institute of Pharmacology and Toxicology, University of Zurich, Zurich, Switzerland; 2 Neuroscience Center Zurich, University and ETH Zurich, Zurich, Switzerland; 3 Department of Neurogenetics, Max Planck Institute for Multidisciplinary Sciences, Göttingen, Germany; 4 Solomon H. Snyder Department of Neuroscience, Johns Hopkins University, Baltimore, MD USA; 5 Centro de Estudios Científicos (CECs), Valdivia, Chile; 6 Facultad de Medicina y Ciencia, Universidad San Sebastián, Valdivia, Chile

**Keywords:** Oligodendrocyte, Molecular neuroscience

## Abstract

The integrity of myelinated axons relies on homeostatic support from oligodendrocytes (OLs). To determine how OLs detect axonal spiking and how rapid axon–OL metabolic coupling is regulated in the white matter, we studied activity-dependent calcium (Ca^2+^) and metabolite fluxes in the mouse optic nerve. We show that fast axonal spiking triggers Ca^2+^ signaling and glycolysis in OLs. OLs detect axonal activity through increases in extracellular potassium (K^+^) concentrations and activation of Kir4.1 channels, thereby regulating metabolite supply to axons. Both pharmacological inhibition and OL-specific inactivation of Kir4.1 reduce the activity-induced axonal lactate surge. Mice lacking oligodendroglial Kir4.1 exhibit lower resting lactate levels and altered glucose metabolism in axons. These early deficits in axonal energy metabolism are associated with late-onset axonopathy. Our findings reveal that OLs detect fast axonal spiking through K^+^ signaling, making acute metabolic coupling possible and adjusting the axon–OL metabolic unit to promote axonal health.

## Abstract (web summary)

Looser et al. reveal that oligodendrocytes detect and respond to axonal activity using K^+^ signaling to regulate vital metabolic support and maintain axon health.

## Main

Oligodendrocytes (OLs) produce and maintain the myelin sheaths around axons, making fast and economical communication between distant neurons possible. Axonal health is crucial for brain function, and axonal damage is a feature of aging and various neurological disorders^1,2^. Accumulating evidence reveals that, apart from orchestrating axonal signaling speed, OLs have an important role in preserving neural circuits and long-term neuronal integrity^3–7^. In recent years, several studies have indicated that OLs contribute to supporting axonal energy metabolism^8–10^. OLs can sustain their functions through aerobic glycolysis alone, given the preservation of white matter integrity in *Cox10*-mutant mice, in which mitochondrial respiration is specifically perturbed in OLs^8^. One necessary outcome of aerobic glycolysis is the production of lactate, which could serve as an energy substrate for axons^8,9,11,12^. Indeed, OL-specific deletion of monocarboxylate transporter 1 (MCT1) leads to late-onset axonopathy, implying that lactate and/or pyruvate release from OLs has a role in axonal health^9,13^. Glutamatergic signaling has been shown to stimulate the surface expression of glucose transporter 1 (GLUT1) in OLs, suggesting that axonal activity might regulate the metabolic support provided by OLs to axons^10^. Metabolite supply could be facilitated by cytosolic channels within the myelin sheath^14,15^, and a disruption in this myelinic channel system has been associated with axonal damage^16^. Moreover, mice deficient in the myelin proteolipid protein (PLP), a mouse model of spastic paraplegia, develop severe axonal spheroids with age^17,18^, possibly due to deficits in axonal transport^19^, alterations in mitochondrial function^20^ and impaired energy homeostasis^21^. Other homeostatic functions carried out by OLs include antioxidant support^22^ and K^+^ buffering^23,24^.

Despite the existing notion that OLs support axonal energy metabolism, the molecular and cellular events involved in metabolic coupling remain elusive. Whether neuronal activity influences OLs to drive metabolic support is still unclear. Glutamatergic signaling may mediate the long-term adjustment of oligodendroglial glucose uptake capacity^10^, but what controls a rapid and on-demand delivery of metabolites to axons remains unexplored.

Independent of neuronal subtype, a key indicator of axonal activity is transient increases in extracellular K^+^ concentrations ([K^+^]_ext_), which depolarize the plasma membrane of OLs^25–27^. Here, we hypothesized that activity-driven K^+^ signaling triggers rapid metabolic coupling between OLs and axons. We addressed this question through optic nerve electrophysiology and two-photon imaging, a combination previously used to study axonal ATP dynamics^11,28^. We found that high-frequency axonal spiking triggers a Ca^2+^ surge and immediately accelerates glucose consumption in OLs. Axonal activity is detected by OLs predominantly through increases in [K^+^]_ext_ and activation of Kir4.1 channels. Both high-frequency stimulation and elevated [K^+^]_ext_ evoke a lactate increase in axons, which is diminished by pharmacological inhibition of Kir4.1. Moreover, blocking Kir4.1 impairs the recovery of axonal firing from high-frequency stimulation. Using OL-specific Kir4.1-knockout mice (*Kir4.1*^*fl/fl*^*;MOGiCre*, hereafter termed Kir4.1 cKO), we demonstrate that axonal lactate dynamics are controlled by oligodendroglial Kir4.1 and that OLs are the primary cells involved in activity-dependent K^+^ clearance. Furthermore, axonal glucose uptake and consumption are decreased in Kir4.1 cKO mice, revealing that OLs also regulate axonal glucose metabolism. These early deficits in axonal energy metabolism could affect vesicular transport and antioxidant capacity, leading to the late-onset axonal damage detected in Kir4.1 cKO mice. Our findings imply that increased [K^+^]_ext_ during fast axonal spiking stimulates axon–OL metabolic coupling and that oligodendroglial K^+^ homeostasis regulates axonal energy metabolism, function and survival.

## Results

### Axonal spiking triggers Ca^2+^ signaling and glycolysis in OLs

**Fig. 1:**
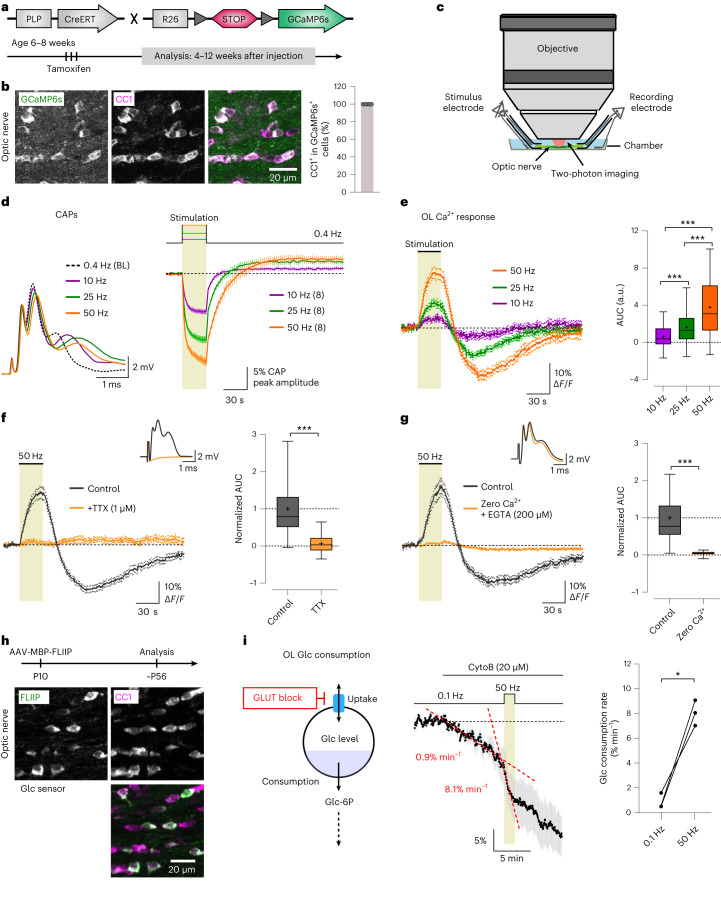
Axonal activity-induced Ca^2+^ signaling and glycolytic flux in optic nerve OLs. **a**, Generation and tamoxifen treatment of *PLP-CreERT;RCL-GCaMP6s* mice at 6–8 weeks; experiments were performed 4–12 weeks after injection. **b**, Left, immunohistochemistry images of GCaMP6s expression in OLs (anti-green fluorescent protein (anti-GFP) antibody, green; anti-CC1 antibody, magenta). Right, percentage of GCaMP6s-positive cells that are also positive for CC1 (*n* = 4 mice, gray circles). **c**, Optic nerve preparation for electrophysiology and imaging. **d**, Left, example CAPs at 0.4 Hz (baseline (BL)) and after 30-s stimulation at 10, 25 or 50 Hz. Right, time course (mean ± s.e.m.) of CAP peak 2 amplitude relative to baseline (*n* = 8 mice). **e**, Left, OL Ca^2+^ responses (mean ± s.e.m.) to different axonal stimulations. Right, box plots showing the response area under the curve (AUC; *n* = 108 cells from eight mice). Higher frequencies induced larger Ca^2+^ surges (50 versus 25 Hz, ***P < 0.0001; 50 versus 10 Hz, ***P < 0.0001; 25 versus 10 Hz, ***P < 0.0001; one-way analysis of variance (ANOVA), Tukey’s multiple-comparison test). **f**, Left, TTX (1 μM) abolished the 50-Hz-induced OL Ca^2+^ response. Inset, CAP diminished by TTX. Right, normalized response AUCs before and after TTX (*n* = 82 cells from six mice), showing a reduction by 93 ± 9% (***P < 0.0001, two-sided paired *t* test). **g**, Left, removal of extracellular Ca^2+^ (+200 μM EGTA) diminished the OL Ca^2+^ response.Inset, CAP response in zero Ca^2+^. Right, normalized response AUCs showing a 96 ± 14% reduction (*n* = 44 cells from three mice, ***P < 0.0001, two-sided paired *t* test). **h**, AAV-mediated glucose (Glc) FRET sensor (FLIIP) expression in adult optic nerve OLs following intracerebroventricular injection. Immunostaining with CC1 (magenta) confirmed FLIIP expression (anti-GFP antibody, green) in mature OLs (observed in three mice). MBP, myelin basic protein; P10, postnatal day 10; ∼P56, approximately postnatal day 56. **i**, Left, schematic of glucose consumption after inhibiting glucose uptake with the glucose transporter (GLUT) blocker CytoB. Glc-6P, glucose 6-phosphate. Middle, time course of glucose decline during CytoB incubation at 0.1 Hz and upon transient 50-Hz stimulation (mean ± s.e.m.). The mean decline rate (red dashed lines) increased from 0.9 ± 0.4% min^−1^ at 0.1 Hz to 8.1 ± 0.6% min^−1^ at 50 Hz. Right, graph showing the glucose consumption rates (*n* = 3 mice, *P = 0.0159, two-sided paired *t* test). Box plots show the median (center line), quartiles (box bounds), mean (+) and 5th–95th percentiles (whiskers).

To investigate Ca^2+^ dynamics in mature OLs as a function of electrical activity, we used *PLP-CreERT* mice^29^ crossed with Ai96 mice expressing the cytosolic Ca^2+^ indicator GCaMP6s in a Cre-dependent manner (*RCL-GCaMP6s*)^30,31^. We studied 3- to 5-month-old *PLP-CreERT;RCL-GCaMP6s* mice treated with tamoxifen at 6–8 weeks (Fig. 1a). We confirmed through immunohistochemistry (Table 1) that GCaMP6s expression was restricted to mature (CC1-immunopositive) OLs (Fig. 1b). We focused on the optic nerve, a myelinated white matter tract ideal for recording compound action potentials (CAPs) and for two-photon sensor imaging^28^ (Fig. 1c). To determine whether mature OLs detect axonal spiking, we stimulated optic nerves at 10, 25 or 50 Hz for 30 s. Before and after this period, nerves received 0.4-Hz electrical pulses to monitor CAP changes alongside OL Ca^2+^ imaging. CAP peak amplitude decreased during high-frequency stimulation (Fig. 1d). Notably, axonal stimulation induced a biphasic Ca^2+^ response in OL somas, marked by an initial Ca^2+^ increase during stimulation and a transient undershoot after stimulation (Fig. 1e and Supplementary Video 1). The Ca^2+^ response was significantly larger at higher frequencies (Fig. 1e). Tetrodotoxin (TTX, 1 µM) application abrogated the stimulus-induced Ca^2+^ surge in OLs (Fig. 1f), confirming the necessity of axonal spiking. Removal of extracellular Ca^2+^ also diminished the OL Ca^2+^ response (Fig. 1g), indicating a mechanism involving Ca^2+^ influx.

**Extended Data Fig. 1:**
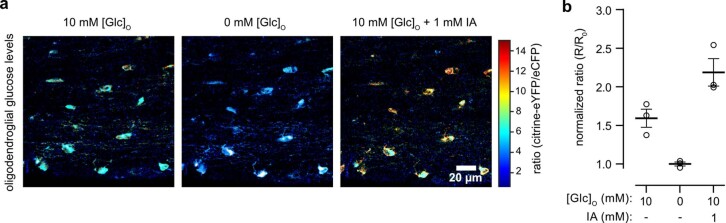
Functional assessment of glucose sensor in OLs. **a**, AAV-mediated glucose FRET sensor FLIIP expression in optic nerve OLs of wildtype mice. Depicted are representative color-coded ratio images from optic nerve in ACSF containing 10 mM glucose (Glc), after removal of extracellular Glc, and following inhibition of glycolysis by 1 mM iodoacetate (IA) in ACSF containing 10 mM Glc. Warm and cold colors indicate high and low ratios or glucose levels, respectively. Scalebar 20 µm. **b**, Quantification of normalized ratios obtained from conditions presented in **a**. Ratios (n = 3 mice) were normalized to the averaged minimum obtained at 0 mM [Glc]_O_. Data shown in mean ± SEM.

Recognizing that OLs detect axonal spiking, we next asked whether heightened axonal activity influences metabolic flux within OLs. Considering that OLs may supply axons with glycolytic products such as pyruvate or lactate, increased axonal spiking could enhance glucose consumption in OLs. To study this, we expressed the glucose sensor FLII12Pglu700μΔ6 (FLIIP)^32^ in optic nerve OLs of wild-type mice through adeno-associated virus (AAV) delivery (Fig. 1h,i). CC1 immunolabeling confirmed sensor expression in mature OLs (Fig. 1h). We first assessed the sensor’s response to glucose fluctuations. Removing extracellular glucose lowered cytosolic glucose levels in OLs, whereas blocking glycolysis with 1 mM iodoacetate (IA) in artificial cerebrospinal fluid (ACSF) containing 10 mM glucose increased glucose levels (Extended Data Fig. 1a,b), confirming the sensor’s functionality for studying glucose metabolism in OLs. To analyze glycolytic flux, we inhibited glucose transporters with 20 µM cytochalasin B (CytoB) and measured the glucose decline rate (Fig. 1i), as previously outlined^33,34^. Remarkably, transient 50-Hz axonal stimulation accelerated oligodendroglial glucose consumption by approximately ninefold compared to the basal rate at 0.1-Hz stimulation (Fig. 1i). Thus, OLs respond to axonal spiking by promptly increasing their glycolytic activity.

### OLs detect axonal spiking through [K^+^]_ext_ and Kir4.1 channels

**Extended Data Fig. 2:**
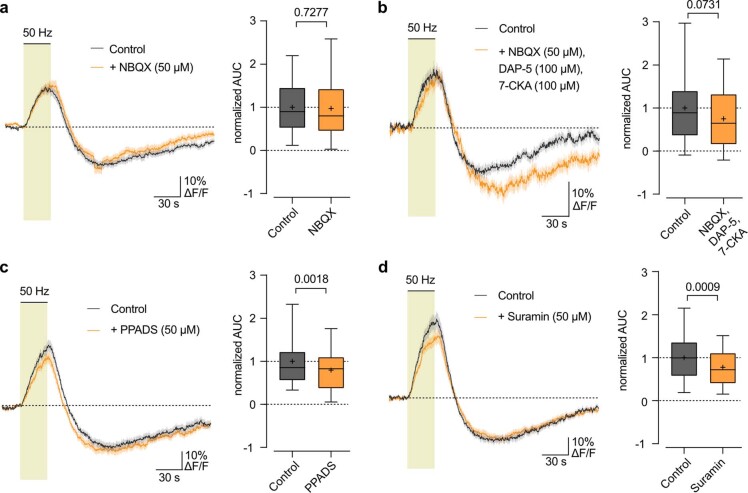
Minor contribution of glutamatergic and purinergic signaling to stimulus-evoked Ca^2+^ response in OLs. **a**, 50 Hz-evoked OL soma Ca^2+^ changes in control condition and with addition of NBQX (50 µM). Quantification of OL Ca^2+^ surge (AUC during stimulation period, boxplots) revealed no difference in the stimulus-evoked Ca^2+^ increase (n = 45 cells from 3 mice, p = 0.7277, two-sided paired t-test). **b**, OL Ca^2+^ changes with NBQX (50 µM), DAP-5 (100 µM), and 7-CKA (100 µM) revealed a decrease in the stimulus-evoked Ca^2+^ increase by 25 ± 13% (n = 33 cells from 3 mice, p = 0.0732, two-sided paired t-test). **c**, OL Ca^2+^ response with PPADS (50 µM) revealed a decrease in the stimulus-evoked Ca^2+^ increase by 21 ± 6% (n = 46 cells from 3 mice, p = 0.0018, two-sided paired t-test). **d**, OL Ca^2+^ response with Suramin (50 µM) revealed a decrease in the stimulus-evoked Ca^2+^ increase by 22 ± 6% (n = 33 cells from 2 mice, p = 0.0009, two-sided paired t-test). Traces represent mean ± SEM. Boxplots show median (line), quartiles (box bounds), mean (+), and 5-95 percentiles (whiskers).

Next, we sought to determine the mechanism by which OLs detect axonal spiking. OLs express *N*-methyl-d-aspartate (NMDA) receptors, which regulate oligodendroglial glucose import^10^ and mediate Ca^2+^ increase in myelin upon electrical axonal stimulation^35^ or chemical ischemia^36^. α-Amino-3-hydroxy-5-methyl-4-isoxazolepropionic acid (AMPA) receptors were also suggested to influence myelinic Ca^2+^ dynamics^35^. Therefore, we examined the contribution of glutamatergic signaling. Blocking AMPA receptors with 2,3-dioxo-6-nitro-7-sulfamoyl-benzo[*f*]quinoxaline (NBQX; 50 µM) caused no change in the OL Ca^2+^ response (Extended Data Fig. 2a), indicating that AMPA receptors might not be involved. Additional inhibition of NMDA receptors with 7-chlorokynurenic acid (7-CKA; 100 µM) and d-2-amino-5-phosphonopentanoate (D-AP5; 100 µM), blocking both glycinergic and glutamatergic NMDA receptor binding sites, reduced the Ca^2+^ response by approximately 20% (Extended Data Fig. 2b). We then examined purinergic signaling, given that ATP may act as a signaling molecule in the white matter and OLs express P2X/P2Y receptors^37–40^. The broad-spectrum, nonselective P2X/P2Y receptor antagonists pyridoxalphosphate-6-azophenyl-2′,4′-disulfonic acid (PPADS; 50 µM) and suramin (50 µM) reduced the Ca^2+^ response by 20% (Extended Data Fig. 2c,d). Hence, both purinergic and glutamatergic signaling modestly contribute to the OL Ca^2+^ response.

**Fig. 2:**
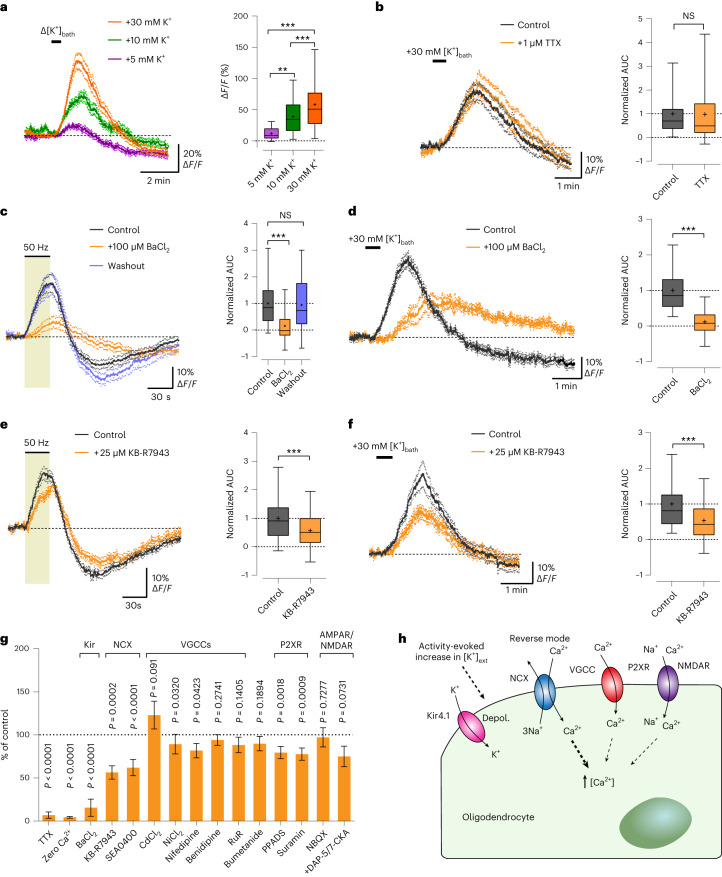
Kir4.1 channel-mediated mechanism underlying stimulus-evoked Ca^2+^ signaling in OLs. **a**, OL Ca^2+^ levels increased by increasing [K^+^]_ext_ with 5, 10 and 30 mM K^+^ (30-s bath application: Δ[K^+^]_bath_). Left, average OL Ca^2+^ traces (mean ± s.e.m.). Right, quantification of Δ[K^+^]_bath_-evoked signal amplitudes (30 mM: *n* = 57 cells from four mice; 10 mM: *n* = 52 cells from five mice; 5 mM: *n* = 35 cells from four mice; 5 versus 10 mM, **P = 0.0048; 5 versus 30 mM, ***P < 0.0001; 10 versus 30 mM, ***P < 0.0001; one-way ANOVA with Tukey’s multiple-comparison test). **b**, Left, K^+^-evoked OL Ca^2+^ response independent of axonal spiking activity, showing comparable surges with TTX. Right, box plots showing the normalized response AUCs (*n* = 72 cells from five mice; *P* = 0.8144, two-sided paired *t* test; NS, not significant). **c**, Left, barium (Ba^2+^, 100 µM) reversibly inhibited the 50-Hz-induced OL Ca^2+^ surge by 84 ± 10%. Right, box plots showing the normalized response AUCs (*n* = 45 cells from four mice; ***P < 0.0001, two-sided paired *t* test). **d**, Left, Ba^2+^ reduced the K^+^-evoked OL Ca^2+^ response by 88 ± 9%. Right, box plots showing the normalized response AUCs (*n* = 47 cells from three mice; ***P < 0.0001, two-sided paired *t* test). **e**,**f**, Reverse-mode NCX blocker KB-R7943 (25 μM) reduced the 50-Hz-induced Ca^2+^ increase (**e**) by 44 ± 11% (*n* = 64 cells from five mice; paired *t* test, ***P = 0.0002) and the K^+^-evoked Ca^2+^ response (**f**) by 47 ± 8% (*n* = 52 cells from three mice; two-sided paired *t* test, ***P < 0.0001). Box plots on the right show the normalized response AUCs. **g**, Summary of drugs tested and their inhibitory effects on 50-Hz-evoked OL Ca^2+^ surges (data are also shown as box plots including the respective *P* values in **c** and **e**, Fig. 1f,g, and Extended Data Figs. 2, 4 and 5): TTX (*n* = 82), zero Ca^2+^ (*n* = 44), BaCl_2_ (*n* = 45), KB-R7943 (*n* = 64), SEA0400 (*n* = 54), CdCl_2_ (*n* = 54), NiCl_2_ (*n* = 60), nifedipine (*n* = 39), benidipine (*n* = 56), RuR (*n* = 71), bumetanide (*n* = 77), PPADS (*n* = 46), suramin (*n* = 33), NBQX (*n* = 45) and +DAP-5/7-CKA (*n* = 33). AMPAR, AMPA receptor; NMDAR, NMDA receptor. **h**, Schematic of axonal activity-mediated OL Ca^2+^ activation: high-frequency axonal activity increases [K^+^]_ext_, depolarizing (Depol.) OLs through Kir4.1 and enhancing Ca^2+^ entry through NCX. Minor contributions of VGCCs, P2XR and NMDA receptors are illustrated. Box plots in **a**–**f** show the median (center line), quartiles (box bounds), mean (+) and 5th–95th percentiles (whiskers).

**Extended Data Fig. 3:**
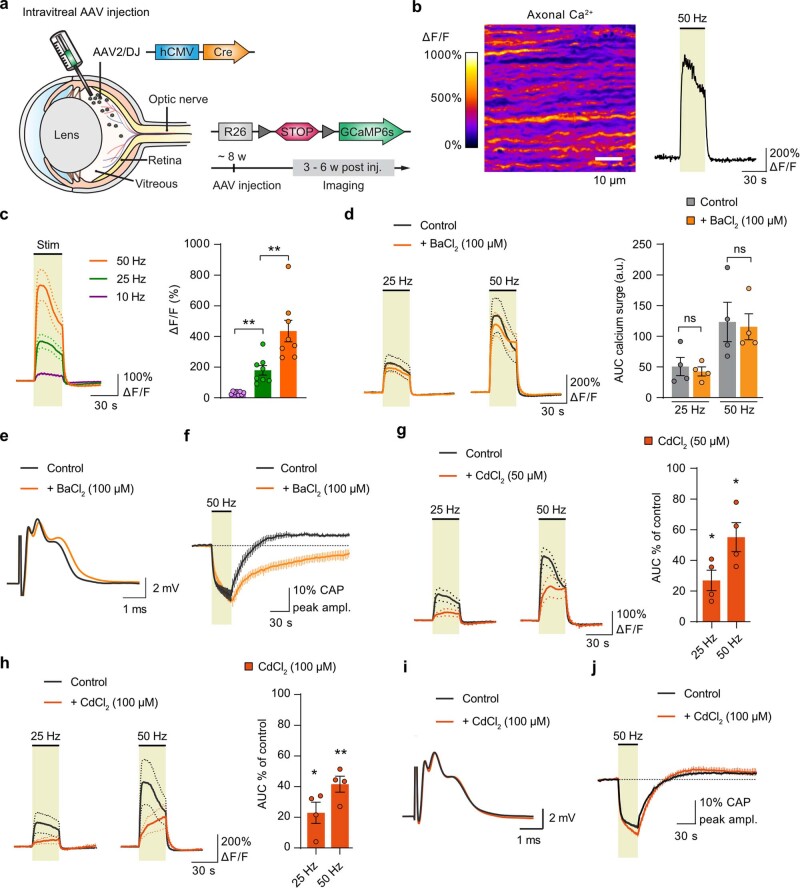
Stimulus-evoked axonal Ca^2+^ surge not sensitive to barium. **a**, Intravitreal AAV-Cre injection into ∼8 weeks old *RCL-GCaMP6s* mice drives GCaMP6s expression in optic nerve axons and used for two-photon imaging. **b**, Electrical 50 Hz stimulation triggers Ca^2+^ rise in axons. Depicted is an example recording of a 30 s stimulation, with ΔF/F image (left) and corresponding Ca^2+^ trace. **c**, Stimulus-evoked axonal Ca^2+^ transients are significantly larger with higher stimulation frequencies (n = 8 mice; 10 Hz vs 25 Hz, p = 0.0027; 25 Hz vs 50 Hz, p = 0.0013; one-way ANOVA with Tukey’s multiple comparisons test). **d**, Stimulus-evoked axonal Ca^2+^ surge is not affected by application of 100 µM Ba^2+^, tested at 25 Hz (n = 4 mice, p = 0.49, two-sided paired t-test) and 50 Hz (n = 4, p = 0.6859, two-sided paired t-test). **e**, Example CAP trace in control conditions and with addition of 100 µM Ba^2+^. **f**, Time course of CAP peak amplitude changes upon 50 Hz stimulations. Note that the recovery kinetics of the peak amplitude are strongly reduced in the presence of Ba^2+^ compared to control (n = 4 mice, *F*_*interaction*_ (74, 444) = 2.487, p < 0.0001, two-way ANOVA). **g** and **h**, Inhibition of VGCCs with Cd^2+^ significantly reduced the stimulus-evoked axonal Ca^2+^ surge. At 50 µM Cd^2+^ (**g**) the 25 Hz- and 50 Hz-evoked Ca^2+^ surges were reduced to 27 ± 7% (n = 4 mice; p = 0.0133, two-sided paired t-test) and to 55 ± 9% (n = 4 mice; p = 0.0343, two-sided paired t-test), respectively. At 100 µM Cd^2+^ (**h**) the 25 Hz- and 50 Hz-evoked Ca^2+^ surges were reduced to 23 ± 7% (n = 4 mice; p = 0.0402, two-sided paired t-test) and by 42 ± 5% (n = 4 mice; p = 0.0074, two-sided paired t-test), respectively. **i**, Example CAP trace in control conditions and with addition of 50 µM Cd^2+^. **j**, CAP recovery kinetics after 50 Hz stimulation is not affected by Cd^2+^ (n = 4 mice). Data are represented as mean time traces ± SEM and as dot-plots with means ± SEM.

Given that axonal action potentials increase [K^+^]_ext_ in the white matter^23,41,42^, we tested whether K^+^ is the key signal responsible for OL stimulation. Transient increases in the bath [K^+^] ([K^+^]_bath_) induced a Ca^2+^ response in OLs, which was more pronounced at higher K^+^ levels (Fig. 2a). We ruled out that increasing [K^+^]_bath_ may indirectly stimulate axonal firing and possible neurotransmitter release, as, in the presence of TTX, OLs showed the same [K^+^]_bath_-evoked Ca^2+^ response (Fig. 2b). OLs express Kir4.1 (refs. ^23,24,43^), implicated in [K^+^]_ext_ homeostasis and OL depolarization^23,26,27,43^. Strikingly, blocking Kir4.1 channels with 100 µM barium (Ba^2+^) reversibly reduced the evoked OL Ca^2+^ response by 80% (Fig. 2c). Ba^2+^ also inhibited the [K^+^]_bath_-evoked Ca^2+^ response in OLs (Fig. 2d), implying that Kir4.1-mediated OL depolarization mediates the Ca^2+^ surge. We then examined whether Ba^2+^ influences axonal Ca^2+^ dynamics. For this, we performed intravitreal delivery of AAV containing Cre in *RCL-GCaMP6s* mice, followed by two-photon imaging of GCaMP6s-expressing optic nerve axons 3–6 weeks after injection (Extended Data Fig. 3a). Electrical stimulation elicited a strong Ca^2+^ increase in axons (Extended Data Fig. 3b and Supplementary Video 2), which was larger at higher frequencies (Extended Data Fig. 3c). Crucially, 100 µM Ba^2+^ did not affect the axonal Ca^2+^ surge (Extended Data Fig. 3d), emphasizing that the Ba^2+^-mediated reduction in the OL Ca^2+^ response (Fig. 2c) was not due to unspecific inhibition of axonal Ca^2+^ signaling. Yet, Ba^2+^ affected the recovery of axonal firing after 50-Hz stimulation, as shown by the delayed recovery of the CAP peak amplitude (Extended Data Fig. 3e,f).

**Extended Data Fig. 4:**
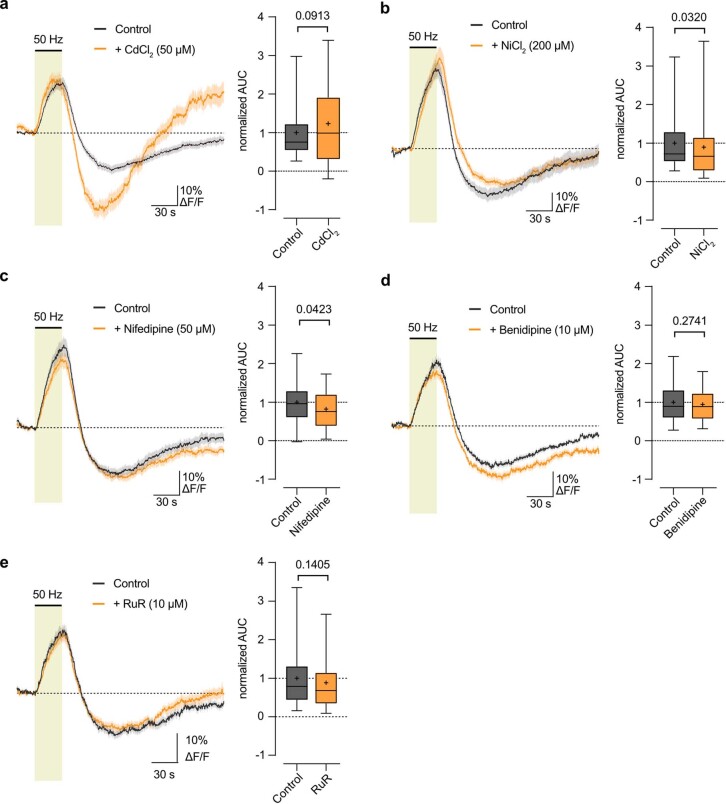
Voltage-gated Ca^2+^ channels are no major drivers of stimulus-evoked OL Ca^2+^ response. **a**, OL Ca^2+^ response in control condition and with Cd^2+^ (50 µM) revealed a slight increase in the 50 Hz-evoked Ca^2+^ rise (n = 54 cells from 4 mice, p = 0.0913 two-sided paired t-test). **b**, OL Ca^2+^ response with Ni^2+^ (200 µM) revealed a slight decrease in the 50 Hz-evoked Ca^2+^ surge by 11 ± 5% (n = 60 cells from 4 mice, p = 0.0320 two-sided paired t-test). **c**, OL Ca^2+^ response with Nifedipine (50 µM) revealed a slight decrease in the 50 Hz-evoked Ca^2+^ surge by 18 ± 9% (n = 39 cells from 3 mice, p = 0.0423 two-sided paired t-test). **d**, OL Ca^2+^ response with Benidipine (10 µM) revealed no overt change in the 50 Hz-evoked Ca^2+^ surge (n = 56 cells from 3 mice, p = 0.2741 two-sided paired t-test). **e**, OL Ca^2+^ response with RuR (10 µM) revealed no significant changes in the 50 Hz-evoked Ca^2+^ surge (n = 71 cells from 4 mice, p = 0.1405 two-sided paired t-test). Traces represent mean ± SEM. Boxplots show median (line), quartiles (box bounds), mean (+), and 5-95 percentiles (whiskers).

OL depolarization could activate voltage-gated Ca^2+^ channels (VGCCs). We tested various VGCC blockers, including cadmium (Cd^2+^), nickel (Ni^2+^), nifedipine, benidipine and ruthenium red (RuR; Extended Data Fig. 4a–e), of which only Ni^2+^ and nifedipine had minimal effects (Extended Data Fig. 4b,c). In contrast, Cd^2+^ did not affect the OL Ca^2+^ surge (Extended Data Fig. 4a) but clearly reduced the stimulus-evoked axonal Ca^2+^ response (Extended Data Fig. 3g,h). This suggests that the axonal Ca^2+^ increase during electrical activity is mediated by VGCCs, unlike the OL Ca^2+^ response. Moreover, the delayed recovery of CAP conductance after high-frequency stimulation was specific to Ba^2+^ (Extended Data Fig. 3f) and not influenced by Cd^2+^ (Extended Data Fig. 3i,j).

**Extended Data Fig. 5:**
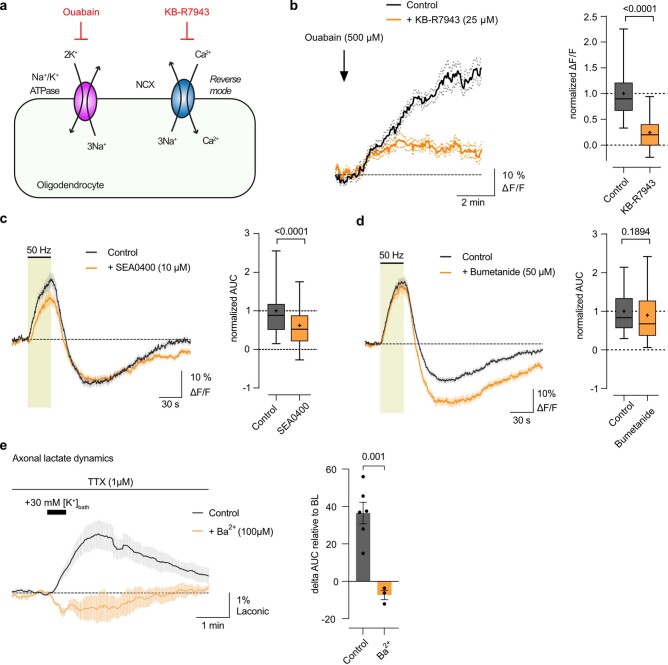
Reverse-mode NCX activation in OL Ca^2+^ response and extracellular K^+^-induced lactate surge in axons. **a**, Schematic of reverse-mode NCX activity testing in OLs: Blocking the sodium pump with Ouabain raises intracellular Na^+^ concentration, which should activate NCX to exchange Na^+^ out for Ca^2+^ in. The ouabain-evoked Ca^2+^ is expected to be reduced by blocking NCX with KB-R7943. **b**, Indeed, 500 µM ouabain application resulted in increased Ca^2+^ levels in OLs, significantly reduced with 25 µM KB-R7943 (n = 31 cells from 2 mice, p < 0.0001, two-sided paired t-test). **c**, 50 Hz-evoked OL Ca^2+^ response with NCX blocker SEA0400 (10 µM) revealed a significant decrease in Ca^2+^ surge by 38 ± 7% (n = 54 cells from 4 mice, p < 0.0001, two-sided paired t-test). **d**, OL Ca^2+^ response with NKCC1 blocker Bumetanide (50 µM) revealed no difference in the stimulus-evoked Ca^2+^ surge (n = 77 cells from 4 mice, p = 0.1894, two-sided paired t-test). **b-d**, Ca^2+^ traces represent means ± SEM. Boxplots show data median as line, upper and lower quartile as bounds of box, mean as + and 5-95 percentiles as whiskers. **e**, Axonal lactate levels following transient 30 mM extracellular [K^+^] increase via 30 s bath application. Experiments were conducted in 1 µM TTX to inhibit axonal activity. Lactate levels are presented as % changes (± SEM), and the lactate surge (delta AUC) is compared to baseline (BL) before K^+^ application. The K^+^-induced axonal lactate rise (n = 6 mice) was abolished in the presence of 100 µM Ba^2+^ (n = 3 mice, p = 0.001, two-sided Student’s t-test).

OLs express Na^+^/Ca^2+^ exchangers (NCX)^44–46^, which could allow Ca^2+^ entry in reverse mode upon membrane depolarization^46,47^. Inhibiting sodium pumps with 500 µM ouabain caused a Ca^2+^ surge in OLs, which was reduced by blocking the reverse-mode activity of NCX with 25 µM KB-R7943 (Extended Data Fig. 5a,b), confirming NCX functionality in optic nerve OLs. Further, KB-R7943 reduced the 50-Hz-evoked OL Ca^2+^ response by 45% (Fig. 2e), a result mirrored by 10 µM SEA0400, another NCX blocker (Extended Data Fig. 5c). KB-R7943 also decreased the [K^+^]_bath_-evoked Ca^2+^ response in OLs (Fig. 2f), suggesting that K^+^-induced depolarization of OLs leads to Ca^2+^ entry through reverse-mode NCX.

Na^+^/K^+^/Cl^−^ cotransporter 1 (NKCC1) is expressed in developing OLs^48,49^ and may have a role in the volume regulation of the axon-facing inner tongue^48^. We tested whether Na^+^/K^+^/Cl^−^ cotransporters are involved in the evoked OL Ca^2+^ surge by using bumetanide, a specific NKCC1 blocker. However, 50 µM bumetanide did not affect the 50-Hz-induced OL Ca^2+^ increase (Extended Data Fig. 5d), suggesting that NKCC1-mediated volume changes in adult OLs are not involved during high-frequency axonal firing.

In summary, our pharmacological results (summarized in Fig. 2g) indicate that OLs detect high-frequency axonal activity through elevated [K^+^]_ext_, leading to depolarization through Kir4.1 channels and Ca^2+^ entry chiefly by reverse-mode activation of NCX (Fig. 2h).

### Oligodendroglial Kir4.1 regulates axonal lactate dynamics

**Fig. 3:**
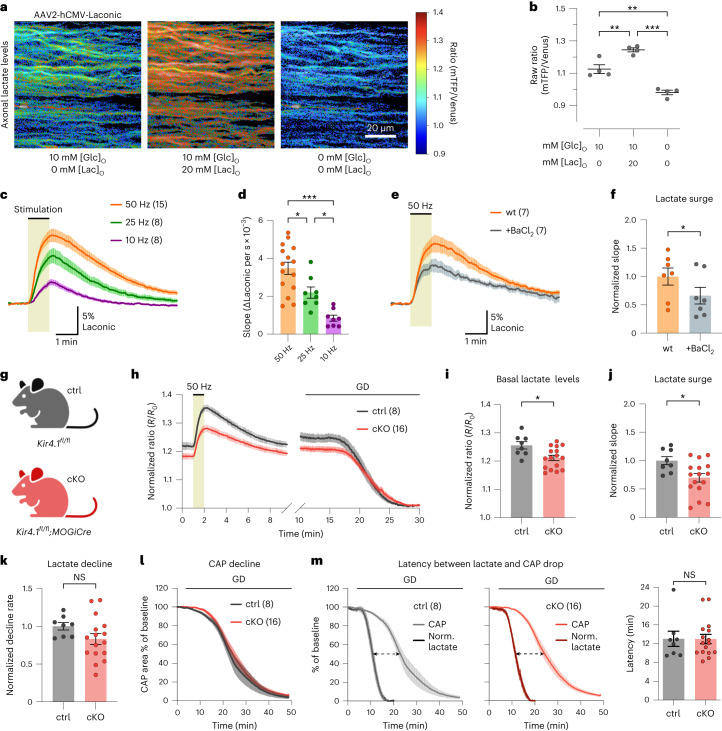
Axonal lactate dynamics are regulated by oligodendroglial Kir4.1. **a**, Lactate FRET sensor (Laconic) expression in optic nerve axons through intravitreal AAV delivery in wild-type mice. Color-coded ratio images in ACSF with 10 mM glucose, additional 20 mM lactate (Lac), and after glucose and lactate removal. hCMV, human cytomegalovirus; mTFP, monomeric teal fluorescent protein. **b**, Ratio quantification of conditions in **a**, confirming the sensor’s response to lactate availability (*n* = 4 mice; [glucose]/[lactate] in mM: 10/0 versus 10/20: **P = 0.0052; 10/0 versus 0/0: **P = 0.0014; 10/20 versus 0/0: ***P < 0.0001; one-way ANOVA Holm–Šídák’s multiple-comparison test). **c**, Lactate level changes (%) upon 10-Hz (*n* = 8 mice), 25-Hz (*n* = 8 mice) or 50-Hz (*n* = 15 mice) stimulation. **d**, Lactate surges (initial slopes), showing higher lactate increases at higher frequencies (50 Hz, *n* = 15 mice; 25 Hz, *n* = 8 mice; 10 Hz, *n* = 8 mice; 50 versus 25 Hz, *P = 0.0141; 50 versus 10 Hz, ***P < 0.0001; 25 versus 10 Hz, *P = 0.0141; one-way ANOVA with Holm–Šídák’s multiple-comparison test). **e**,**f**, Ba^2+^ (100 μM) reduced the 50-Hz-induced axonal lactate increase (**e**) by 34 ± 11% (**f**) (*n* = 7, two-sided paired *t* test, *P = 0.0249). wt, wild type. **g**, OL-specific Kir4.1 cKO (*Kir4.1*^*fl/fl*^*;MOGiCre*) and control (ctrl) mice (*Kir4.1*^*fl/fl*^). **h**–**m**, Axonal lactate and CAP analyses in ∼3-month-old cKO (*n* = 16) and control (*n* = 8) mice. **h**, Time course of axonal lactate levels during 50-Hz stimulation and GD. Traces were normalized to the minimum level after GD. **i**, Basal axonal lactate levels were lower in cKO versus control (*P = 0.0119, two-sided unpaired *t* test). **j**, 50-Hz-evoked lactate surge was lower in cKO (*P = 0.0174, two-sided unpaired *t* test). **k**, Lactate decline rate during GD was similar between genotypes (*P* = 0.1318, two-sided unpaired *t* test). **l**, CAP decline kinetics during GD were comparable between genotypes (CAP decline slope: *P* = 0.7934, time to 50% CAP area: *P* = 0.7265, two-sided unpaired *t* test). **m**, Normalized (Norm.) axonal lactate and CAP time traces during GD in control (left) and cKO (middle) mice. Right, graph showing that the temporal delay between 50% lactate decline and CAP drop (dashed arrows in left and middle) was similar between genotypes (*P* = 0.9789, two-sided unpaired *t* test). Data are represented as mean ± s.e.m.

Given that OLs respond to fast axonal spiking through Kir4.1 channel activation, we speculated that this K^+^-driven stimulation could regulate metabolite supply to axons (for example, on-demand lactate delivery). To test this, we expressed the lactate sensor Laconic^50^ in optic nerve axons using intravitreal AAV delivery (Fig. 3a). Initial tests in wild-type nerves showed that increasing extracellular lactate levels increased axonal lactate levels (Fig. 3a,b), confirming the expression of lactate transporters in axons. Removal of glucose and lactate from the ACSF significantly reduced axonal lactate levels (Fig. 3a,b). Hence, the Laconic sensor is not saturated at baseline, and its dynamic range allows for studying axonal lactate dynamics. Notably, axonal lactate levels increased during high-frequency spiking, more prominently at higher stimulation frequencies (Fig. 3c,d). To see whether elevated [K^+^]_ext_ alone could increase axonal lactate independently of spiking, we used TTX to inhibit axonal activity and the associated workload increase. Indeed, [K^+^]_bath_ stimulation increased axonal lactate levels, which was mediated by Kir4.1 activity (Extended Data Fig. 5e). Importantly, Kir4.1 inhibition with Ba^2+^ specifically diminished the stimulus-evoked OL Ca^2+^ response but did not affect the axonal Ca^2+^ surge (Fig. 2c and Extended Data Fig. 3d) and thus the axonal workload upon spiking. Yet, Ba^2+^ reduced the activity-induced increase in axonal lactate levels by 40% (Fig. 3e,f). This implies that K^+^-mediated axon–OL signaling facilitates lactate supply to axons during active spiking.

**Extended Data Fig. 6:**
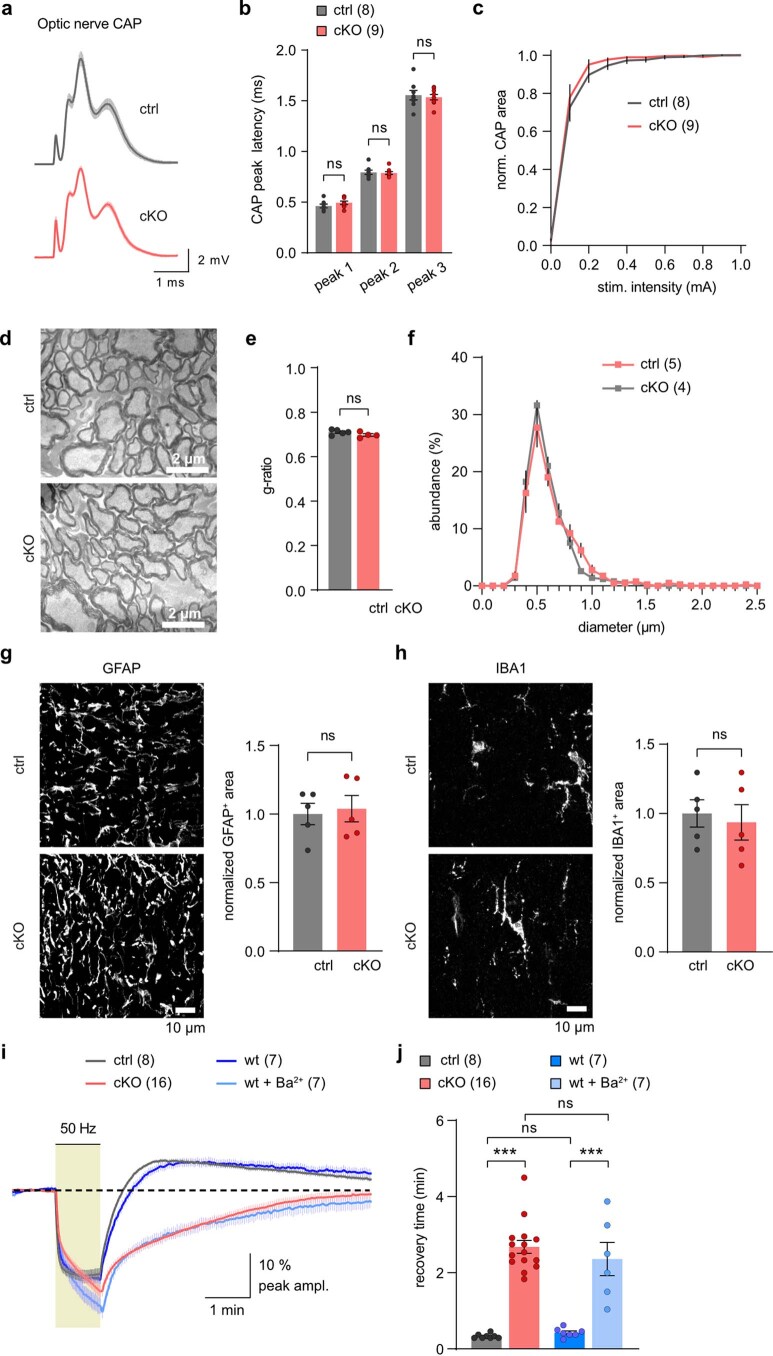
Oligodendroglial Kir4.1 critical for white matter K^+^ clearance. **a**, Average optic nerve CAP response of ctrl (n = 8) and Kir4.1 cKO (n = 9) mice. **b**, Similar CAP peak latencies between genotypes (ctrl n = 8, cKO n = 9; p = 07637 for peak 1, p = 0.9958 for peak 2, p = 0.9265 for peak 3, one-way ANOVA with Holm-Šídák’s –multiple comparisons test). **c**, Similar stimulus-response relationships in both groups (ctrl n = 8, cKO n = 9; *F*_*interaction*_ (10, 150) = 0.4445, p = 0.9224, two-way ANOVA). CAP area from each stimulus intensity normalized to max stimulation at 1 mA. **d-f**, Electron microscopic (EM) analysis of optic nerves from 3-months-old cKO (n = 4) and ctrl (n = 5) mice: **d**, Representative EM images. **e**, Myelin sheath thickness (g-ratio) comparable between genotypes (p = 0.1584, two-sided Student’s t-test). **f**, Similar axon size distribution of myelinated axons (*F*_*interaction*_ (25, 175) = 1.028, p = 0.4333, two-way ANOVA). **g** and **h**, Confocal images of optic nerve immunolabeling: **g**, GFAP; **h**, IBA1, in 2.5-months-old ctrl and cKO mice. No differences in GFAP-immunopositive area between genotypes (ctrl, n = 5; cKO n = 5; p = 0.7611, two-sided Student’s t-test), or IBA1 labeling (ctrl, n = 5; cKO n = 5; p = 0.7047, two-sided Student’s t-test). **i**, Averaged CAP amplitude changes (% from baseline) upon 1 min 50 Hz stimulation of optic nerves from cKO, ctrl, wildtype (wt) and wt treated with 100 µM Ba^2+^ (wt+Ba^2+^). **j**, CAP peak recovery time post 50 Hz stimulation: Slower recovery in cKO (n = 16 mice) and wt+Ba^2+^ (n = 7) compared to ctrl (n = 8) and wt (n = 7) (ctrl vs cKO, p < 0.0001; wt vs wt+Ba^2+^, p < 0.0001; ctrl vs wt, p = 0.9970; cKO vs wt+Ba^2+^, p = 0.7362; one-way ANOVA with Holm-Šídák’s multiple comparisons test). Data represented as means ± SEM.

**Extended Data Fig. 7:**
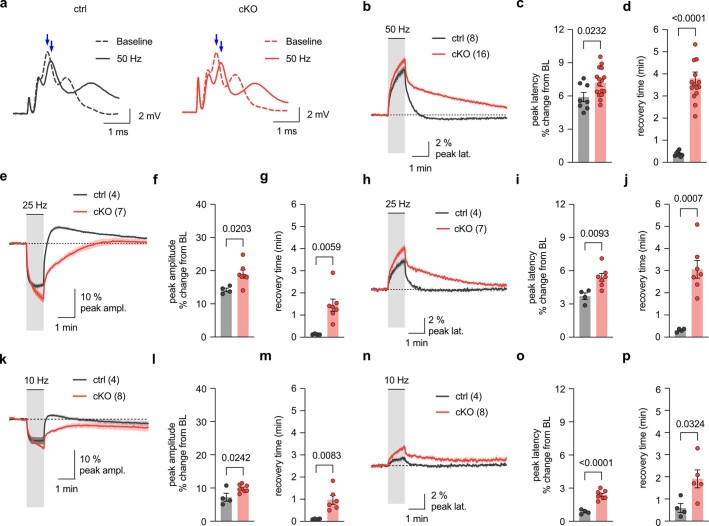
Altered CAP peak latency recovery kinetics in Kir4.1 cKO. **a**, Representative CAP traces of ctrl and cKO at baseline and post 50 Hz stimulus train, highlighting changes in CAP peak amplitude and latency (blue arrows). **b-d**, Analysis of CAP peak latency changes to 1 min 50 Hz in ctrl (n = 8 mice) and cKO (n = 16 mice): **b**, Average % changes (± SEM) from baseline in CAP peak latency showing slower recovery in cKO. **c**, CAP peak latency analysis at the end the stimulation revealing larger increase in latency in cKO compared to ctrl (p = 0.0232, two-sided Student’s t-test). **d**, Analysis of CAP peak latency recovery time post stimulation, significantly slower in cKO compared to ctrl (p < 0.0001, two-sided Student’s t-test). **e-j**, 25 Hz-induced CAP changes in ctrl (n = 4 mice) and cKO (n = 7 mice). **e-g**: CAP peak amplitude: **e**, Average % changes in peak amplitude. **f**, Greater decrease in cKO (p = 0.0203, two-sided Student’s t-test). **g**, Slower recovery post-stimulation in cKO (p = 0.0059, two-sided Student’s t-test). **h-j**, CAP peak latency: **h**, Average % changes in latency. **i**, Larger increase in cKO mice (p = 0.0093, two-sided Student’s t-test). **j**, Slower recovery post-stimulation in cKO (p = 0.0007, two-sided Student’s t-test). **k-p**, 10 Hz-induced CAP changes in ctrl (n = 4 mice) and cKO (n = 8 mice). **k-m**, CAP peak amplitude: **k**, Average % changes in peak amplitude. **l**, Greater decrease in cKO mice (p = 0.0242, two-sided Student’s t-test). **m**, Slower recovery post-stimulation in cKO (p = 0.0083, two-sided Student’s t-test). **n-p**, CAP peak latency: **n**, Average % changes in latency. **o**, Larger increase in cKO mice (p < 0.0001, two-sided Student’s t-test). **p**, Slower recovery post-stimulation in cKO (p = 0.0324, two-sided Student’s t-test). Data are represented as dot-plots with means ± SEM.

To investigate whether oligodendroglial Kir4.1 specifically controls axonal lactate dynamics, we used Kir4.1 cKO mice^23^ (Fig. 3g and Extended Data Fig. 6). First, we inspected optic nerves from ∼3-month-old Kir4.1 cKO mice and littermate controls (*Kir4.1*^*fl/fl*^). We found no differences in CAP peak latencies or nerve excitability (Extended Data Fig. 6a–c). Additionally, we observed no overt changes in myelin sheath thickness and the diameter distribution of myelinated axons (Extended Data Fig. 6d–f). At this age, no signs of axonal damage or neuroinflammation were observed (Extended Data Fig. 6d,g,h). The Ba^2+^-mediated impact on delaying CAP peak recovery (Extended Data Fig. 3f) could involve Kir4.1-mediated K^+^ clearance by OLs and/or astrocytes^43,51^. Notably, Kir4.1 cKO nerves showed significantly slower kinetics of CAP peak recovery after high-frequency stimulation compared to littermate controls (Extended Data Fig. 6i,j), confirming earlier results^23^. Interestingly, wild-type nerves treated with 100 µM Ba^2+^ showed the same recovery delays as Kir4.1 cKO nerves (Extended Data Fig. 6i,j), implying that oligodendroglial Kir4.1 is primarily involved in K^+^ buffering in the adult white matter, with little to no contribution of astrocytic Kir4.1 or other Ba^2+^-sensitive Kir channels. The recovery kinetics of CAP peak latency were also affected in Kir4.1 cKO nerves (Extended Data Fig. 7a–d). Poststimulation CAP recovery deficits were also visible at 25 and 10 Hz (Extended Data Fig. 7e–p), demonstrating that oligodendroglial Kir4.1 also governs activity-dependent K^+^ clearance at lower frequencies.

We then examined axonal lactate dynamics and found that both the resting lactate levels (at 0.1-Hz stimulation) and the high-frequency-evoked lactate surge were notably reduced in Kir4.1 cKO nerves compared to controls (Fig. 3h–j). Basal axonal lactate levels were compared after normalizing the Förster resonance energy transfer (FRET) ratios to minimal lactate levels after glucose deprivation (GD; Fig. 3h,i). Lower lactate levels in Kir4.1 cKO axons might stem from decreased OL lactate supply, altered axonal glycolysis or heightened lactate consumption. The latter seems unlikely, as lactate decay during GD was not faster but marginally slower in cKO mice than in controls (Fig. 3h,k). This is further corroborated by a similar CAP decline rate during GD (Fig. 3l). The two genotypes showed the same latency between lactate depletion and CAP decline (Fig. 3m). Notably, the CAP drop was observed only when axonal lactate neared depletion (Fig. 3m). Given the concurrent decline of CAP and ATP levels during GD^11^, this suggests that axonal lactate fuels ATP for action potentials and axons cease firing as lactate supply dwindles, leading to CAP decline (Fig. 3m). This also implies comparable axonal mitochondrial respiration between the genotypes.

### Reduced MCT1 and GLUT1 in central nervous system myelin of Kir4.1 cKO mice

**Extended Data Fig. 8:**
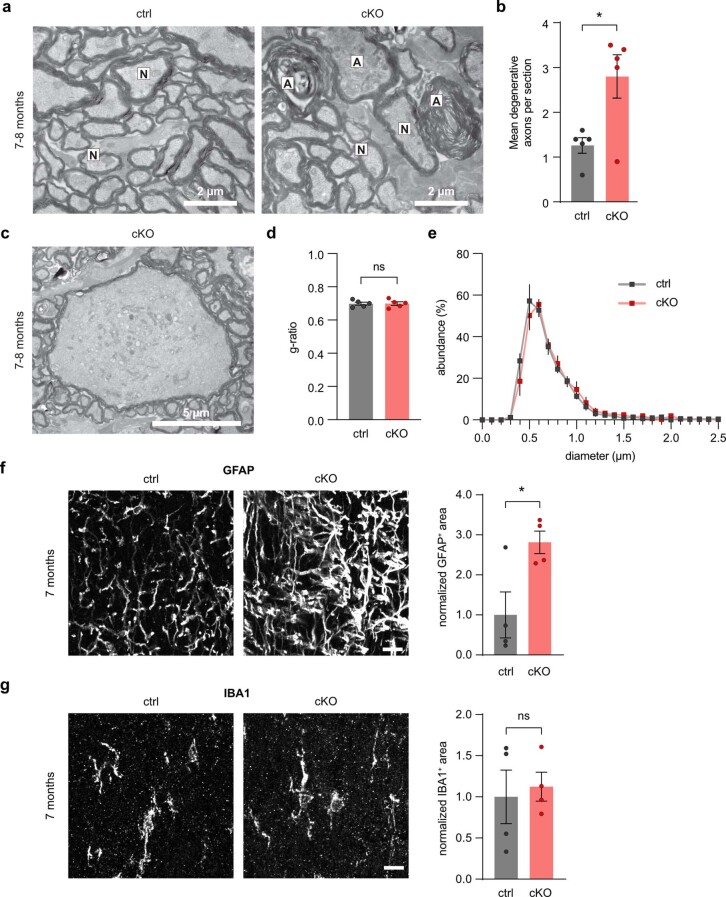
Age-related axonopathy and signs of astrogliosis in 7- to 8-months-old Kir4.1 cKO mice. **a-c**, EM analysis of optic nerves from 7- to 8-months-old cKO and ctrl mice: **a**, Representative EM images with normal (N) appearing axons and signs of axonopathy (A). Scalebar 2 µm. **b**, Ultrastructural features of axonal injury and degeneration more frequent in cKO compared to ctrl (n = 5 mice per group, with 10 randomly taken images each covering 286 µm (ref. ^2^); p = 0.0169, two-sided Student’s t-test). **c**, Notably, giant axonal swellings were exclusive to cKO nerves at this age. Scalebar 5 µm. **d**, No difference in myelin sheath thickness (g-ratio) between genotypes (n = 5 mice; p = 0.9813, two-sided Student’s t-test). **e**, Similar axon size distribution of myelinated axons (n = 5 mice; *F*_*interaction*_ (27, 216) = 0.6975, p = 0.8670, two-way ANOVA). **f**, Significant increase in GFAP-immunopositive area, indicative of astrogliosis, in optic nerves from cKO mice compared to ctrl at 7 months of age (n = 4 mice; p = 0.0295, two-sided Student’s t-test). Scalebar 10 µm. **g**, No significant differences in IBA1-immunopositive area between genotypes (n = 4 mice; p = 0.7493, two-sided Student’s t-test). Scalebar 10 µm. Data are represented as means ± SEM.

Loss of oligodendroglial Kir4.1 may affect axonal integrity with age^24^. Electron microscopy (EM) at age 3 months revealed no signs of impaired axonal integrity in Kir4.1 cKO mice, with normal myelin thickness and axonal diameters (Extended Data Fig. 6d–f). However, by 7–8 months, there was a significant increase in axon/myelin profiles indicative of axonal degeneration, including giant axonal swellings (Extended Data Fig. 8a–c). The age-dependent axonopathy in cKO mice occurred without noticeable myelin abnormalities or thinning (Extended Data Fig. 8d,e). Additionally, increased glial fibrillary acidic protein (GFAP) immunolabeling was observed, consistent with secondary astrocytic reactivity; however, there was no increase in ionized calcium-binding adaptor molecule 1 (IBA1) immunopositivity, suggesting no microgliosis (Extended Data Fig. 8f,g).

**Fig. 4:**
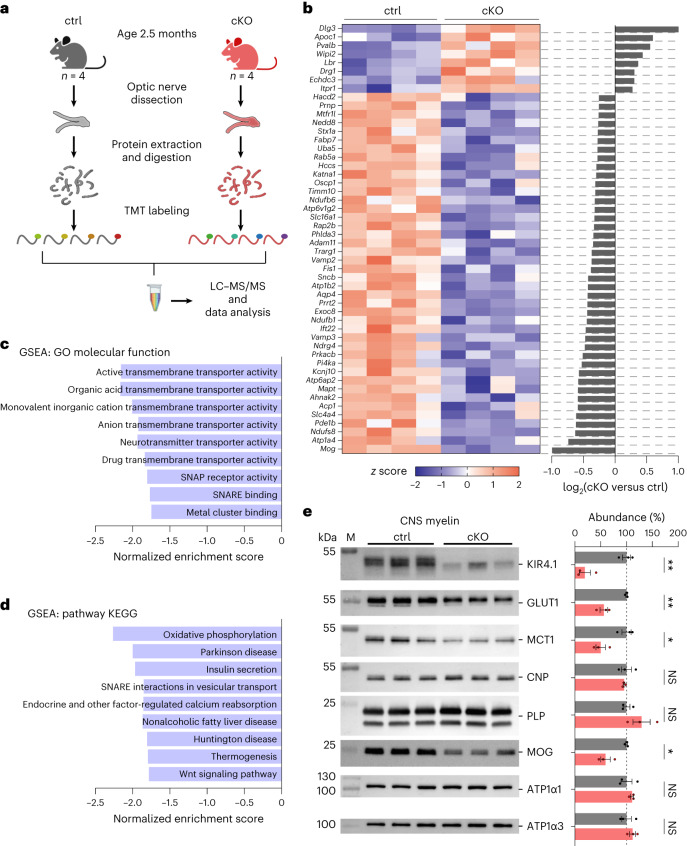
MCT1 and GLUT1 levels are reduced in central nervous system myelin of Kir4.1 cKO mice. **a**, TMT-based proteomics analysis in optic nerves from 2.5-month-old Kir4.1 cKO (*n* = 4) and littermate control (*n* = 4) mice. The scheme (generated by BioRender) shows extraction, digestion, TMT labeling and pooling for liquid chromatography–tandem mass spectrometry (LC–MS/MS). **b**, Top 50 (sorted by FDR) differentially regulated proteins listed with gene names. The heat map shows upregulation (red) or downregulation (blue) in cKO versus control ranked by log_2_(fold change), including only proteins with fold change >0.25 or <−0.25. Row *z* scores were calculated from normalized intensities. **c**,**d**, Gene set enrichment analyses (GSEAs) for the categories Gene Ontology (GO) molecular function (**c**) and pathway Kyoto Encyclopedia of Genes and Genomes (KEGG) (**d**) showed decreases in transmembrane transporter activity, vesicular transport and energy metabolism (FDR < 0.05). Analysis was performed through WebGestalt.org, with proteins ranked by log_2_(fold change). SNAP, soluble *N*-ethylmaleimide-sensitive factor attachment protein; SNARE, SNAP receptor. **e**, Left, immunoblot analysis of Kir4.1, MCT1 and GLUT1 in myelin biochemically purified from the brains of 2.5-month-old control (*n* = 3) and cKO (*n* = 3) mice. M, molecular weight marker. Right, compared to controls (gray), cKO mice (red) showed a reduced abundance of Kir4.1 by 81 ± 13% (**P = 0.0038), GLUT1 by 44 ± 8% (**P = 0.0044) and MCT1 by 50 ± 13% (*P = 0.0179, two-sided unpaired *t* test). Known myelin proteins PLP, 2′,3′-cyclic nucleotide 3′-phosphodiesterase (CNP), myelin oligodendrocyte glycoprotein (MOG), ATPase Na^+^/K^+^ transporting subunit α1 (ATP1α1) and ATP1α3 were detected as markers. Note that the MOG abundance was reduced by 40 ± 9% (*P = 0.0125, two-sided unpaired *t* test), attributed to Cre insertion under the MOG promoter in heterozygous *MOGiCre* mice inactivating one *mog* allele. Data are represented as mean ± s.e.m.

**Extended Data Fig. 9:**
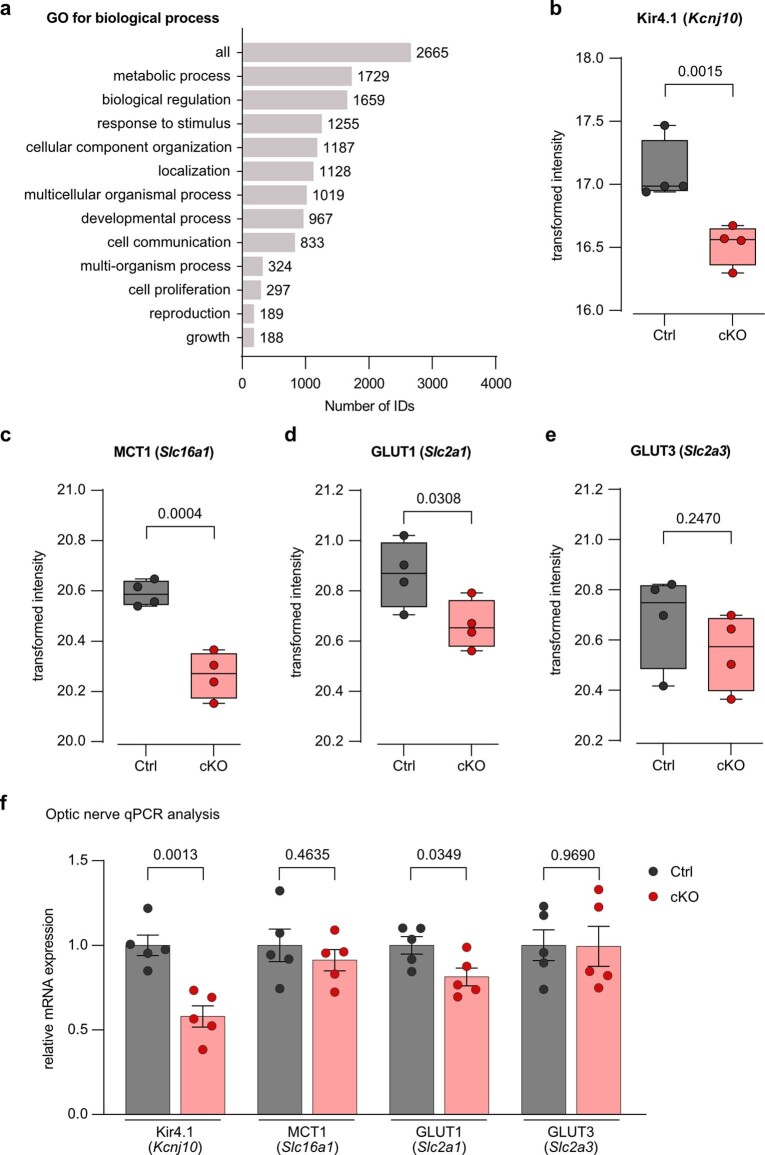
Proteomics and qPCR analysis of optic nerve lysates from young Kir4.1 cKO mice. **a**, Of the 3624 detected protein hits from the TMT-based proteomics analysis (see Fig. 4), 2665 were unambiguously mapped to unique Entrez gene IDs. Using the GO term biological process, bar charts depict the GO annotation and functional categorization of identified proteins. WebGestalt.org provided the summary. **b**, Protein abundance of Kir4.1 (gene *Kcnj10*) is reduced in samples from cKO (n = 4 mice) compared to ctrl (n = 4, p = 0.0015, moderated t-test). **c**, Abundance of MCT1 (*Slc16a1*) is reduced in cKO compared to ctrl (n = 4, p = 0.0004, moderated t-test). **d**, Abundance of GLUT1 (*Slc2a1*) is reduced in cKO compared to ctrl (n = 4, p = 0.0308, moderated t-test). **e**, Abundance of GLUT3 (*Slc2a3*) is unchanged between genotypes (n = 4, p = 0.2470, moderated t-test). Boxplots with all points show median (line), quartiles (box bounds), and min to max (whiskers). **f**, Relative mRNA abundance in optic nerve lysates of 3-months-old cKO (n = 5) and littermate ctrls (n = 5): Compared to ctrl, Kir4.1 mRNA levels were reduced by 0.42 ± 0.09 (p = 0.0013, two-sided Student’s t-test) and GLUT1 mRNA levels were reduced by 0.19 ± 0.07 (p = 0.0349, two-sided Student’s t-test). No significant differences in mRNA levels of MCT1 (p = 0.4635) and GLUT3 (p = 0.9690). Data represented as dot-plots with means ± SEM.

The absence of oligodendroglial Kir4.1 in young mice appears to affect axonal energy metabolism before axonal pathology onset. This aligns with the lower axonal lactate levels observed in young cKO nerves, which did not show axonal damage or neuroinflammation. Both pharmacological inhibition and genetic inactivation of Kir4.1 affected the stimulus-evoked axonal lactate surge, but the lower basal lactate level was an additional feature of cKO mice. Besides regulating acute metabolic coupling, oligodendroglial Kir4.1 may also contribute to adjusting the axon–OL metabolic unit. To explore the metabolic changes from Kir4.1 deficiency further, we performed tandem mass tag (TMT)-based quantitative proteomics on optic nerve lysates from 2.5-month-old Kir4.1 cKO mice and littermate controls (Fig. 4a, Extended Data Fig. 9a and Supplementary Data 1). Expectedly, Kir4.1 (*Kcnj10*) protein levels were lower in the cKO group (Fig. 4b and Extended Data Fig. 9b). Among the top 50 proteins sorted by the false discovery rate (FDR), those associated with vesicular transport and energy metabolism showed reduced levels (Fig. 4b). Gene set enrichment and pathway analyses revealed declines in transmembrane transporter activity, vesicular transport and oxidative phosphorylation pathways (Fig. 4c,d). A lower abundance of MCT1 (*Slc16a1*; Fig. 4b and Extended Data Fig. 9c) and GLUT1 (*Slc2a1*; Extended Data Fig. 9d) was observed, but there was no change in the glucose transporter GLUT3 (*Slc2a3*; Extended Data Fig. 9e). Additional qPCR analysis indicated a slight decrease in *Slc2a1* mRNA but not in *Slc16a1* mRNA (Extended Data Fig. 8f), implying that changes in transporter abundance might not be solely due to gene expression alterations. Immunoblotting of myelin purified from cKO brains revealed an approximately 50% reduction in both GLUT1 and MCT1 abundance (Fig. 4e). Hence, Kir4.1 loss affected the relative abundance of metabolite transporters in OLs. K^+^ and Kir4.1-mediated signaling could influence gene expression, protein synthesis, surface trafficking and/or turnover of metabolite transporters in OLs. This might result in adaptations aligning the support machinery of OLs to axonal activity levels.

### Minor changes in axonal ATP dynamics in Kir4.1 cKO mice

**Fig. 5:**
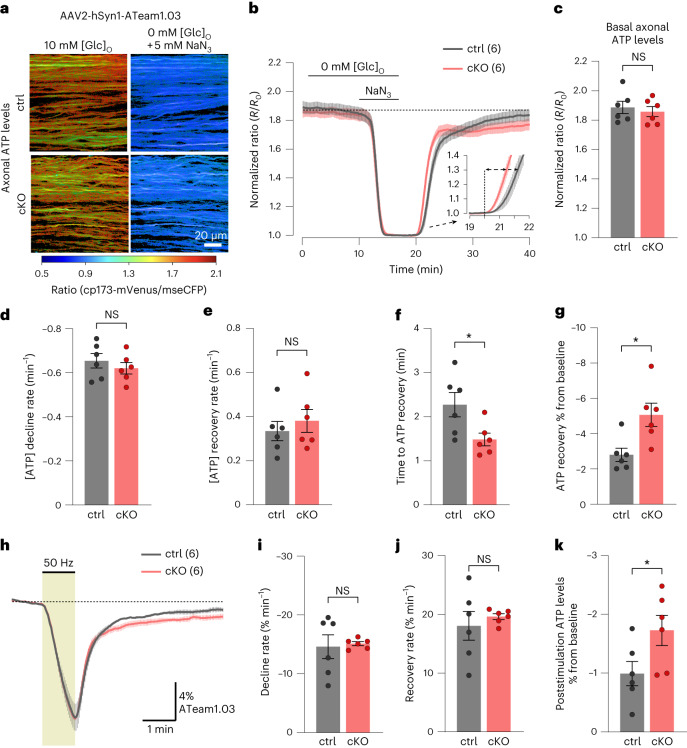
Minor changes in axonal ATP dynamics in the absence of oligodendroglial Kir4.1. **a**, AAV-mediated ATP FRET sensor (ATeam1.03) expression in optic nerve axons. Color-coded ratio images from control (top) and cKO (bottom) nerves show ATP levels in ACSF with 10 mM glucose and after GD + MI with 5 mM NaN_3_. hSyn1, human synapsin 1; mseCFP, monomeric superenhanced cyan fluorescent protein. **b**, Time course of axonal ATP levels in ∼3-month-old cKO (*n* = 6) and control (*n* = 6) mice challenged with GD + MI, normalized to the minimum ATP level (*R*_0_). Inset, initial ATP recovery dynamics following reperfusion with 10 mM glucose. **c**, Basal axonal ATP levels were comparable between genotypes (*n* = 6 mice, *P* = 0.61, two-sided unpaired *t* test). **d**, No difference in the ATP decline rate between genotypes upon GD + MI (*n* = 6 mice, *P* = 0.44, two-sided unpaired *t* test). **e**, Similar initial ATP recovery rates between genotypes after GD + MI (*n* = 6 mice, *P* = 0.51, two-sided unpaired *t* test). **f**, Onset of ATP recovery (see dashed arrows in inset in **b**) differed between control and cKO nerves (*n* = 6 mice, *P = 0.03, two-sided unpaired *t* test). **g**, Lower axonal ATP level recovery in cKO than in controls following GD + MI (*n* = 6 mice, *P = 0.0135, two-sided unpaired *t* test). **h**, Axonal ATP level changes (%) at 50-Hz stimulation relative to baseline. **i**,**j**, Similar ATP level decline rates (**i**) during 50-Hz stimulation (*n* = 6 mice, *P* = 0.81, two-sided unpaired *t* test) and equal initial ATP recovery rates (**j**) after stimulation (*P* = 0.54, two-sided unpaired *t* test). **k**, Slightly lower axonal ATP level recovery after stimulation in cKO versus controls (*n* = 6 mice, *P = 0.0498, two-sided unpaired *t* test). Data are represented as mean ± s.e.m.

Proteomics analysis indicated alterations in oxidative phosphorylation pathways, leading us to question whether cKO mice also exhibit changes in axonal ATP homeostasis. To investigate this, we expressed the ATP sensor ATeam1.03 (ref. ^52^) in axons through intravitreal AAV delivery^28^. First, we assessed basal axonal ATP levels by normalizing the FRET ratios at 0.1-Hz stimulation in 10 mM glucose against ratios after GD and mitochondrial respiration inhibition (MI) using 5 mM sodium azide (NaN_3_) to deplete axonal ATP levels (Fig. 5a–c). No genotype difference in basal axonal ATP levels was observed (Fig. 5c). The ATP decline rate during GD + MI was also comparable between genotypes (Fig. 5b,d), and the ATP recovery rate after NaN_3_ washout and 10 mM glucose reperfusion was unaffected (Fig. 5e). Interestingly, cKO nerves showed a marginally quicker onset of ATP recovery compared to controls (Fig. 5b,f); however, after 15–20 min, the ATP levels in control nerves almost completely returned to baseline levels, whereas cKO nerve recovery was incomplete (Fig. 5b,g).

**Extended Data Fig. 10:**
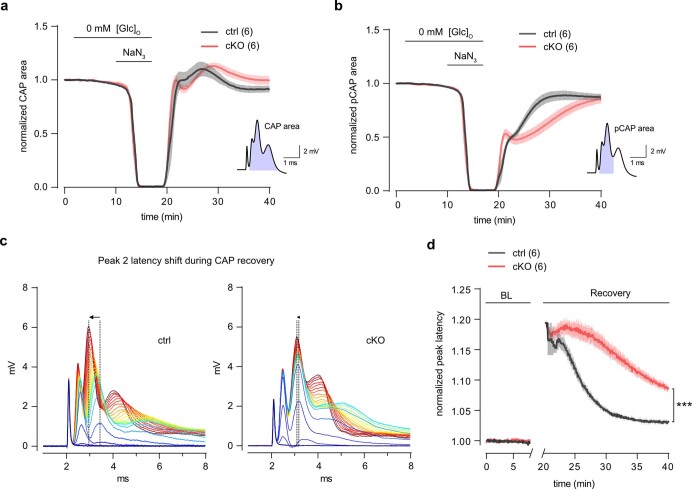
Deficiency in adjusting axonal conduction speed following energy deprivation. **a**, Time course of CAP area changes from optic nerves of ∼3 months old cKO (n = 6) and ctrl mice (n = 6), challenged with glucose deprivation (GD) and mitochondrial inhibition (MI) using 5 mM NaN_3_ (GD + MI, simulating chemical ischemia). See also Fig. 5b. Inset (bottom right) illustrates the CAP area. **b**, Partial CAP (pCAP) area (inset, bottom right) time course analysis during and after GD + MI. Note differing recovery kinetics between genotypes. **c**, Example traces of CAP response recovery (depicted in 25-second intervals, from blue to red), illustrating the first 10 minutes post GD + MI for ctrl (left) and cKO (right). Note the more considerable shift in peak 2 latency (indicated by arrow at dashed lines) during recovery in ctrl compared to cKO. **d**, Peak 2 latency analysis, adjusted to initial baseline value before GD + MI. Notably, post GD + MI, the initial increase in peak 2 latency of the first recovering CAPs was similar in both genotypes; however, its return to normal latency was significantly faster in ctrl than in cKO (n = 6, p < 0.0001, two-way ANOVA). Data represented as means ± SEM.

The minor deficit in ATP recovery prompted us to examine axonal firing recovery in cKO mice further. The rapid decline in the CAP area after GD + MI, indicating axonal conduction block, was comparable between genotypes, and the onset and recovery kinetics of axonal firing appeared unchanged (Extended Data Fig. 10a). The analysis of the partial CAP (pCAP) area, which reflects the dynamics of the first and second CAP peaks^28^, showed comparable decline rates upon GD + MI, whereas pCAP recovery dynamics differed between genotypes (Extended Data Fig. 10b). Closer inspection of CAP waveforms revealed a striking difference in the recovery kinetics of peak latency (Extended Data Fig. 10c). In controls, the CAP peak amplitude increased gradually as the peak latency decreased; however, this peak latency shift (increase in conduction velocity) was markedly reduced in cKO nerves (Extended Data Fig. 10d), indicating a deficit in adjusting conduction speed following acute energy deprivation. Given that chemical ischemia increases [K^+^]_ext_ and affects oligodendroglial K^+^ conductance^53^, K^+^ clearance by OLs upon axon reenergization could be crucial for adjusting conduction speeds. This impaired speed adjustment might contribute to the reduced ATP recovery in cKO axons after chemical ischemia (Fig. 5b,g).

As the recovery of CAP peak latencies after high-frequency stimulation was reduced in cKO mice (Extended Data Fig. 7), we wondered whether this also correlates with axonal ATP recovery. We thus measured ATP dynamics during and after 50-Hz stimulation (Fig. 5h–k). The rate and extent of decreases in axonal ATP levels during high-frequency stimulation were comparable between genotypes (Fig. 5h,i). The initial ATP recovery rate after stimulation was also unchanged (Fig. 5h,j). However, 3–4 min into the recovery phase, cKO axons showed a slightly lower ATP rebound compared to initial baseline values (Fig. 5h,k). Hence, the absence of Kir4.1 and the lower K^+^ clearance rate might increase the energy burden on axons following high-frequency activity or chemical ischemia.

### Reduced axonal glucose metabolism in Kir4.1 cKO mice

**Fig. 6:**
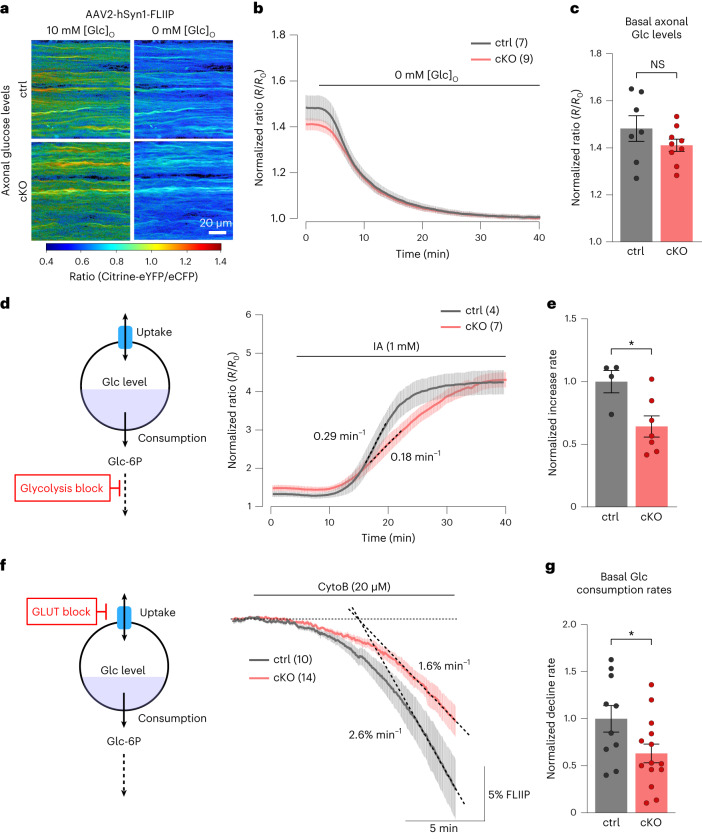
Lack of oligodendroglial Kir4.1 impairs axonal glucose metabolism. **a**, AAV-mediated glucose FRET sensor (FLIIP) expression in optic nerve axons. Color-coded ratio images from control (top) and cKO (bottom) nerves show glucose levels in ACSF with 10 mM glucose and after GD. eYFP, enhanced yellow fluorescent protein; eCFP, enhanced cyan fluorescent protein. **b**, Time course of axonal glucose levels in ∼3-month-old cKO (*n* = 9) and control (*n* = 7) mice during perfusion with regular and zero-glucose ACSF, with 10 mM lactate to sustain CAPs. Traces were normalized to the minimum level after GD. **c**, Comparable basal axonal glucose levels between genotypes (control *n* = 7, cKO *n* = 9, *P* = 0.2276, two-sided unpaired *t* test). **d**,**e**, Glycolysis inhibition (**d**, left) with IA (1 mM) in ACSF with 10 mM glucose increased axonal glucose levels (**d**, right). **e**, Glucose increase rate (dashed lines in **d**) upon IA was lower in cKO (*n* = 7 mice) by 36 ± 13% compared to controls (*n* = 4 mice; *P = 0.0235, two-sided unpaired *t* test). **f**,**g**, Glucose consumption assessed with CytoB (20 µM) during 0.1-Hz stimulation. **f**, Scheme (left) and time course (right) of glucose level decline upon CytoB treatment for control (*n* = 10) and cKO (*n* = 14) mice, with mean decline rates (dashed lines) of 2.6 ± 0.4% min^−1^ and 1.6 ± 0.3% min^−1^, respectively. **g**, Basal axonal glucose consumption rate in cKO mice (*n* = 14) was reduced by 37 ± 17% compared to controls (*n* = 10 mice; *P = 0.0384, two-sided unpaired *t* test). Data are represented as mean ± s.e.m.

The lower axonal lactate levels and activity-induced lactate surges in Kir4.1 cKO mice reflect reduced oligodendroglial metabolic support. Recent findings indicate that, besides lactate, OLs may supply axons with glucose^54^. However, the regulation of glucose uptake and glycolysis in myelinated axons and the potential contribution of OLs remain elusive. To study glucose dynamics, we expressed the glucose sensor FLIIP^32^ in optic nerve axons through intravitreal AAV delivery (Fig. 6a). The basal axonal glucose levels (at 0.1-Hz stimulation) were comparable between genotypes, with basal levels derived by normalizing FRET ratios in ACSF with 10 mM glucose against the minimum levels after GD (Fig. 6a–c). To assess the sensor’s range, we applied IA to inhibit glycolysis, leading to a 250% increase in glucose levels in both genotypes (Fig. 6d). However, the rate of axonal glucose increase was 36% slower in cKO nerves than in controls (Fig. 6d,e), hinting at a lower glucose uptake or hexokinase activity. Measuring glycolytic flux using CytoB, we found a 37% reduction in axonal glucose consumption in cKO nerves (Fig. 6f,g). This implies a similarly decreased glucose uptake in cKO axons to maintain equal basal glucose levels as controls (Fig. 6c), explaining the slower glucose increase with IA treatment (Fig. 6d,e).

**Fig. 7:**
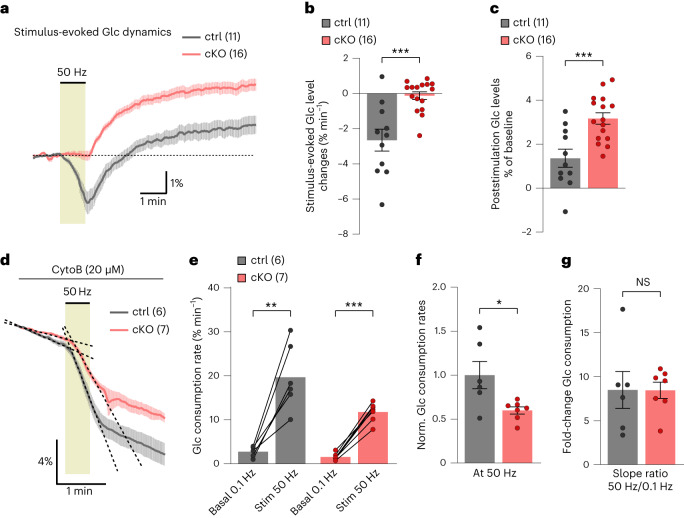
Activity-induced axonal glucose consumption rate is reduced in Kir4.1 cKO mice. **a**, Time-course traces of 50-Hz-evoked axonal glucose dynamics showing differences in glucose level changes between cKO (*n* = 16) and control (*n* = 11) mice. **b**, During stimulation, glucose levels decreased at a rate of 2.7 ± 0.6% min^−1^ in controls (*n* = 11) but remained stable (0.1 ± 0.2% min^−1^) in cKO (*n* = 16; ***P = 0.0002, two-sided unpaired *t* test). **c**, After stimulation, glucose levels increased above the initial baseline values in both genotypes but were significantly higher in cKO (3.2 ± 0.3%) than in controls (1.4 ± 0.4%; ***P = 0.0006, two-sided unpaired *t* test). **d**–**g**, Assessment of glucose consumption rate changes from 0.1- to 50-Hz stimulations in control (*n* = 6) and cKO (*n* = 7) mice. **d**, Decline slopes are indicated by dashed lines. **e**, Axonal glucose consumption rates significantly increased upon 50-Hz stimulation (Stim 50 Hz) in controls (0.1 versus 50 Hz, **P = 0.0022, two-sided paired *t* test) and in cKO (0.1 versus 50 Hz, ***P < 0.0001, two-sided paired *t* test). **f**, Glucose consumption rate during 50-Hz stimulation was 40 ± 15% lower in cKO than in controls (*P = 0.0208, two-sided unpaired *t* test). **g**, Fold change in glucose consumption from 0.1 to 50 Hz was comparable between genotypes (8.5 ± 2 in controls and 8.5 ± 1 in cKO, *P* = 0.9858, two-sided unpaired *t* test). Data are represented as mean ± s.e.m.

While testing whether axonal glucose dynamics differed during 50-Hz stimulation, we observed that glucose levels decreased in control axons but remained steady in cKO axons (Fig. 7a,b). This indicates a stronger activation of glycolysis relative to glucose uptake in control axons but being balanced in cKO axons. After stimulation, glucose levels increased above baseline in both groups (Fig. 7a,c), implying continued glucose uptake activation after stimulation ended. The difference in glucose dynamics during high-frequency activity may result from a lower activation of glucose consumption in cKO compared to controls. Indeed, 50-Hz stimulation increased axonal glucose consumption compared to basal (at 0.1-Hz stimulation) glycolytic activity (Fig. 7d,e), but this increase was 40% less in cKO axons (Fig. 7f). Interestingly, both control and cKO nerves exhibited an eightfold increase in glucose consumption rate under 50-Hz stimulation (Fig. 7g). Therefore, although the glycolytic activation machinery remains intact in cKO axons, their overall glucose metabolism is reduced by approximately 40%, both at rest and during activity. Consequently, we conclude that OLs regulate axonal glucose uptake and consumption, a unique metabolic OL–axon interaction that requires Kir4.1 function.

## Discussion

**Fig. 8:**
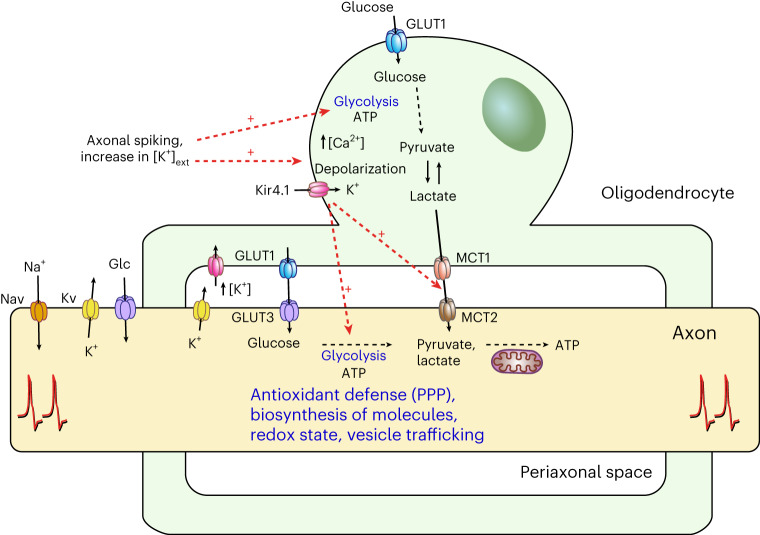
Activity-mediated model of axon–OL metabolic coupling. The scheme shows a working model in which axon–OL communication and metabolic coupling in the white matter are controlled by K^+^ and Kir4.1-mediated signaling. Fast axonal spiking induces a rapid increase in OL [Ca^2+^] and glycolysis. OLs primarily detect axonal activity through elevated [K^+^]_ext_ and activation of Kir4.1 channels. This K^+^-mediated signaling facilitates the supply of lactate (or pyruvate) to axons. Apart from regulating acute metabolic coupling, oligodendroglial Kir4.1 adjusts the myelinic levels of MCT1 and GLUT1. In addition to lactate, OLs might supply axons with glucose and/or modulate axonal glucose uptake at the nodes of Ranvier. Oligodendroglial K^+^ homeostasis also influences axonal glycolysis, which is likely critical for preserving axonal integrity through various glucose metabolism-dependent processes, such as antioxidant protection through the pentose phosphate pathway (PPP), biosynthesis of molecules required for structure and function, regulation of the redox state, and vesicular transport. The potential contribution of astrocytes as a source of (glycogen-derived) lactate (or pyruvate) for axons is not depicted in this scheme, pending future studies. Nav, voltage-gated sodium channel; Kv, voltage-gated potassium channel.

Loss of axonal integrity may result from dysfunctions in the axon–OL unit^5,6,15,22,55–58^. This study underscores the critical role of OLs in maintaining axonal health. We revealed that OLs respond to fast axonal spiking by initiating Ca^2+^ signaling and glycolysis. OLs detect axonal spiking predominantly through [K^+^]_ext_ and Kir4.1 channel activation. This axon–OL signaling mechanism facilitates the supply of metabolites to axons. Disruptions in Kir4.1, whether through pharmacological inhibition or OL-specific inactivation, impaired the activity-induced lactate surges in axons. Lack of oligodendroglial Kir4.1 reduced the myelinic abundance of GLUT1 and MCT1, leading to decreased axonal lactate levels and glucose metabolism. These early metabolic deficits in axons are linked to late-onset axonal damage. We provide a working model in which K^+^ signaling through oligodendroglial Kir4.1 governs metabolic coupling in the white matter (Fig. 8), affecting axonal energy metabolism, function and survival.

Transient increases in [K^+^]_ext_ are a hallmark of axonal activity. OLs, highly permeable to K^+^, depolarize with elevations in [K^+^]_ext_ (ref. ^25^) and axonal spiking^23^, facilitated by Ba^2+^-sensitive Kir channels^26,27^, of which Kir4.1 is predominantly expressed by OLs^23,24,59,60^. Our results suggest that Kir4.1-mediated K^+^ uptake is pivotal in triggering Ca^2+^ influx in OL somas, facilitated by membrane depolarization and reverse-mode NCX activation. This K^+^-driven signaling regulates metabolic coupling between OLs and axons. Other activity signals, such as glutamate, may induce Ca^2+^ activity in microdomains along internodes^61,62^, probably aiding in OL differentiation during development^61,63–67^ and GLUT1 surface expression^10^. In zebrafish, myelin sheaths of new OLs show Ca^2+^ signals in response to neuronal activity^68,69^. However, in developing mouse OLs, Ca^2+^ transients were reported to occur independent of cortical neuron activity^70^. We found that, in mouse optic nerves, Ca^2+^ signaling in mature OLs is elicited chiefly through [K^+^]_ext_ surges during high-frequency axonal firing. K^+^ is released at the nodes of Ranvier^71,72^ and along juxtaparanodal domains under the myelin sheath^73,74^. Activity-mediated [K^+^]_ext_ accumulation around OLs is possibly more pronounced in fully myelinated adult white matter than in sparsely myelinated cortical regions, which merits further investigation. Upon detecting heightened axonal activity, OLs promptly enhance glucose consumption and provide lactate or pyruvate on demand (Fig. 8). This process is likely triggered by [K^+^]_ext_ and Kir4.1 activation. Inhibiting Kir4.1, which specifically disrupts axon–OL signaling, reduced the stimulus- and K^+^-evoked lactate surge in axons. Notably, the pharmacological methods used to evaluate lactate dynamics did not affect axonal activity or workload, which could affect axonal metabolism. Hence, axonal lactate surges involve OL metabolic support, as further evidenced by diminished stimulus-evoked lactate increases in OL-specific Kir4.1 knockouts. [K^+^]_ext_ increases can accelerate glycolysis^34,75^ and promote lactate release through proton-linked monocarboxylate transporters^15^. Hence, a proton increase in OLs, linked to [K^+^]_ext_ (ref. ^53^) and Kir4.1 activity^76^, may facilitate lactate release. Further research is required to clarify a K^+^-driven lactate or pyruvate release mechanism in OLs. Additionally, the contributing role of astrocytes^34,75,77–79^, specifically in the white matter^12,80^, needs more exploration. Embryonic deletion of Kir4.1 from both astrocytes and OL lineage cells causes severe white matter pathology, including myelin vacuole formation^23,51,81,82^. However, the OL lineage-specific deletion of Kir4.1 is devoid of myelin abnormalities^23,24^, which is partly confirmed by this study. Consequently, astrocytic Kir4.1 and K^+^ siphoning, likely involving gap junction coupling to OLs^27,73,81,83^, are critical during white matter development. Yet, in the adult white matter, the glial syncytium appears less critical for K^+^ homeostasis^84^. The CAP recordings in this study suggest that OLs may independently handle the activity-induced K^+^ clearance in adult optic nerves. Beyond acute metabolic coupling, oligodendroglial Kir4.1 may also adjust the support machinery of OLs, indicated by reduced myelinic GLUT1 and MCT1 levels in Kir4.1 cKO mice. Optic nerve proteome analysis revealed a decrease in proteins essential for intracellular membrane trafficking, including vesicle-associated membrane protein 2 (VAMP2), VAMP3, Ras-related protein Rab-2B (RAB2B) and RAB5A, reported to be expressed by mature OLs^85–88^, implicating Kir4.1 in regulating metabolite transporter trafficking^89–91^. Previous research has linked a 50–80% decrease in myelinic MCT1 or GLUT1 levels to late-onset axonal pathology in mice, typically emerging between 8 and 24 months of age^9,10,13^. In this study, Kir4.1 cKO mice showed signs of axonopathy at 7–8 months, preceded by a 50% reduction in both GLUT1 and MCT1 levels at 2–3 months. Moreover, this is associated with early deficits in axonal energy metabolism. In young Kir4.1 cKO mice, axons exhibited lower basal lactate levels and reduced glucose uptake and consumption. These deficits likely result from impaired OL metabolite supply and axonal glucose metabolism. Despite these metabolic changes, axonal ATP homeostasis appeared unaffected. Notably, glucose metabolism is crucial for various cellular functions beyond ATP production^92,93^, including antioxidant protection through the pentose phosphate pathway^94^ and providing glycolytic intermediates for vital structural and functional molecules (Fig. 8). Additionally, axonal glycolysis is key to sustaining fast axonal transport^95^. Hence, impaired axonal glucose metabolism might affect axonal transport and increase vulnerability to oxidative stress, potentially leading to axonal damage with age. The metabolic alterations in the axons of Kir4.1 cKO mice are unlikely due to impaired K^+^ clearance. Extracellular K^+^ accumulation, secondary to defective clearance, should increase axonal workload and stimulate glucose metabolism^96,97^, yet Kir4.1 cKO axons showed decreased glucose metabolism. The primary impact of impaired K^+^ clearance was seen on the speed of CAP peak recovery following high-frequency stimulation, not at basal activity levels. Hence, the reduced lactate levels and glucose metabolism in axons at basal activity are more likely due to deficits in the axon–OL metabolic unit. Furthermore, the unchanged axonal ATP dynamics in cKO mice indicate stable axonal energy demand during spiking. Consequently, the reduced stimulus-evoked lactate and glucose dynamics were not influenced by acute changes in axonal workload. We also found normal mitochondrial respiration in the axons of Kir4.1 cKO mice, with no signs of inflammation or glial activation affecting axonal metabolism at a young age. Notably, this study uncovered that OLs have a crucial role in regulating axonal glucose metabolism, including both uptake and consumption. Experiments in corpus callosum slices have shown that glucose loading in OLs could sustain callosal CAPs in aglycemia conditions, indicating OLs’ ability to transfer glucose to axons^54^. The reduced myelinic GLUT1 abundance, along with reduced glucose uptake in cKO axons, suggests that OLs and myelin affect glucose delivery to axons. The influence of OLs on axonal glycolysis needs further investigation. OLs could affect axonal energy homeostasis through exosome signaling^98,99^. Yet, whether [K^+^]_ext_ and Kir4.1 functions are involved in exosome release, similar to glutamate signaling^100^, remains to be seen. Considering the role of the Na^+^ pump in regulating neuronal energy metabolism^96,97^, adaptations in axonal Na^+^ pumps might influence axonal glycolysis. Conclusively, this study underscores the intricate metabolic interactions between OLs and axons, inviting further exploration into the regulation of axonal glucose metabolism, which could have implications for axonal degeneration in aging and neurodegenerative disease.

## Methods

### Animals

All animal experiments were permitted by the local veterinary authorities in Zurich, in agreement with the guidelines of the Swiss Animal Protection Law, Veterinary Office, Canton Zurich (Animal Welfare Act, December 16, 2005, and Animal Welfare Ordinance, April 23, 2008). *PLP-CreERT;RCL-GCaMP6s* mice were generated by crossing *PLP-CreERT* mice (RRID:IMSR_JAX:005975)^29^ with *ROSA26-floxed-STOP-GCaMP6s* mice (Ai96; RRID:IMSR_JAX:024106)^30^. Heterozygous *RCL-GCaMP6s* (Ai96) mice were used to express GCaMP6s in optic nerve axons following intravitreal delivery of AAV-Cre. Kir4.1 cKO mice^23^ were obtained from crosses of mice carrying the floxed *Kcnj10* (*Kir4.1*^*fl/fl*^)^51^ allele with *MOGiCre* mice^101^. *Kir4.1*^*fl/fl*^ mice were used as littermate controls. Transgenic mouse lines were maintained on the C57BL/6 background. For experiments in wild types, we used Charles River C57BL/6 mice. Both male and female mice were used for experiments. Mice were kept in an inverted 12-h light/dark cycle at 23 °C and 55% humidity. Food and water were available ad libitum.

### Tamoxifen treatment

*PLP-CreERT;RCL-GCaMP6s* mice, aged 6–8 weeks, were treated with tamoxifen (Sigma-Aldrich, T5648). Tamoxifen was freshly prepared in corn oil (Sigma-Aldrich, C8267) at a concentration of 10 mg ml^−1^ for each experimental group. Mice received intraperitoneal injections of 100 mg tamoxifen per kilogram body weight daily for 3 days. Experiments commenced 4 weeks after the initial tamoxifen injection.

### AAV injections

Intravitreal injections were performed following previously published protocols^28^. Anesthesia involved intraperitoneal administration of fentanyl (0.05 mg kg^−1^), midazolam (5 mg kg^−1^) and medetomidine (0.5 mg kg^−1^) in NaCl (0.9%)^102^. Pupil dilation was achieved with topical application of cyclopentolate (1%) and phenylephrine (5%), and Viscotears liquid gel (CIBA Vision) prevented eye dryness. Mice were maintained at 37 °C on a heating pad. Under a SteREO Discovery V20 microscope (Zeiss), a 30-gauge needle (insulin syringe, Omnican 50, Braun) was used to create a scleral incision, through which a 34-gauge Hamilton syringe delivered 1.5 μl of AAV suspension into the vitreous at ∼0.1 µl s^−1^. After injection, ofloxacinum eye drops (Floxal, Bausch + Lomb) were administered, followed by buprenorphine (0.1 mg kg^−1^) treatment after anesthesia reversal with atipamezole (2.5 mg kg^−1^) and flumazenil (0.5 mg kg^−1^). Mice were monitored in low light due to prolonged pupil dilation. Intracerebroventricular AAV injections in P10 pups involved isoflurane anesthesia and a heated stereotaxic frame at 37 °C. Presurgical analgesia included buprenorphine (0.05 mg kg^−1^, subcutaneously administered), lidocaine (10 mg ml^−1^) and bupivacaine (2.5 mg ml^−1^). A 32-gauge Hamilton syringe was used to administer 2 μl of AAV per hemisphere into the ventricle, with coordinates of −2.0 mm anterior–posterior, 0.8 mm medial–lateral and −2.0 mm dorsal–ventral from the bregma, at 500 nl min^−1^. The needle remained in place for 2 min before removal. After surgery, pups received buprenorphine (0.05 mg kg^−1^) and suturing with absorbable, braided Vicryl sutures, with careful monitoring during recovery.

### AAV vectors

The single-stranded (ss) or self-complementary (sc) AAV vectors used in this study were produced, purified and quantified by the viral vector facility of Neuroscience Center Zurich, as previously described^103^. Intravitreal AAV injections were performed with undiluted AAV vectors mixed with fluorescein dye (0.01 mg ml^−1^ in PBS). The green fluorescein dye was added to monitor successful injections into the vitreous. The following AAVs and their physical titers (in vector genomes per milliliter (vg per ml)) were used: to induce GCaMP6s expression in optic nerve axons, we used *RCL-GCaMP6s* (Ai96) mice injected with scAAV-DJ/2-hCMV-chI-Cre-SV40p(A) (3.4 × 10^12^ vg per ml). To study axonal ATP dynamics, we used the FRET sensor ATeam1.03 (ref. ^52^) packaged in ssAAV-2/2-hSyn1-ATeam1.03-WPRE-hGHp(A) (3.0 × 10^12^ vg per ml). Lactate dynamics were studied using the FRET sensor Laconic^50^ packaged in ssAAV-2/2-hCMV-chI-Laconic-WPRE-SV40p(A) (3.0 × 10^12^ vg per ml). Glucose dynamics were studied using the codon-optimized version of the FRET sensor FLIIP^32^ packaged in ssAAV-2/2-hSyn1-FLIIP-WPRE-hGHp(A) (2.9 × 10^12^ vg per ml) or in ssAAV-(1 + 2)/2-MBP-FLIIP-hGHp(A) (1.5 × 10^13^ vg per ml), the latter containing the 1.3-kb MBP promoter previously used to drive reporter gene expression in OLs^104,105^.

### Optic nerve electrophysiology and two-photon imaging

Acute optic nerve preparations for concurrent electrophysiology and two-photon imaging were conducted as previously described^28^. Following isoflurane anesthesia and decapitation, optic nerves were excised and placed in a modified interface perfusion chamber (Haas Top, Harvard Apparatus), perfused with ACSF at 37 °C using a TC-10 temperature control system (NPI Electronic), and continuously oxygenated with 95% O_2_ and 5% CO_2_. Nerve ends were inserted into custom-made suction electrodes filled with ACSF. This setup was integrated with a custom two-photon microscope^106^ featuring a Chameleon Ultra II Ti:sapphire laser (Coherent) and a 25× water immersion objective (XLPLN 25×/1.05 WMP2, Olympus), operated by ScanImage software (r3.8.1, Janelia Research Campus^107^). The nerve was allowed to equilibrate in the perfusion chamber beneath the objective for at least 30 min, ensuring stability. A transistor–transistor logic trigger driven by a stimulus generator (STG4002-1.6mA, Multichannel Systems) was used to synchronize the acquisition of both electrophysiology and imaging data.

### Solutions

Optic nerves were superfused with ACSF containing 126 mM NaCl, 3 mM KCl, 2 mM CaCl_2_, 1.25 mM NaH_2_PO_4_, 26 mM NaHCO_3_, 2 mM MgSO_4_ and 10 mM glucose (pH 7.4), bubbled with 95% O_2_ and 5% CO_2_. For pharmacological interventions, drugs at the concentrations mentioned in the text were added to the ACSF shortly before the experiments. Stock solutions (1,000×) of the following drugs were prepared: TTX (ab120054, Abcam), D-AP5 (0106, Tocris), PPADS (ab120009, Abcam), suramin (1472, Tocris), ouabain (O3125, Sigma-Aldrich), RuR (1439, Tocris), BaCl_2_ (342920, Sigma-Aldrich), CdCl_2_ (202908, Sigma-Aldrich), NiCl_2_ (339350, Sigma-Aldrich), IA (I2512, Sigma-Aldrich), NaN_3_ (S2002, Sigma-Aldrich), NBQX (ab120045, Abcam), 7-CKA (ab120024, Abcam), nifedipine (1075, Tocris), benidipine (3934, Tocris), bumetanide (3108, Tocris), SEA0400 (6164, Tocris), KB-R7943 (ab120284, Abcam) and CytoB (5474, Tocris). When appropriate, drugs were protected from light during preparation and the experiments. For the analysis of stimulus-evoked Ca^2+^ responses, optic nerves were treated with the drugs for 20–30 min before the experiment. For zero-Ca^2+^ experiments, the ACSF contained 200 μM EGTA and 4 mM Mg^2+^ to maintain constant divalent cation concentrations. For [K^+^]_bath_ stimulations, NaCl was adjusted in the ACSF to maintain monovalent cation concentrations. For GD experiments, glucose was substituted with sucrose. Similar sodium and osmolarity adjustments were made when sodium ʟ-lactate (Sigma-Aldrich) was added to the ACSF.

### CAP recordings and analysis

Optic nerves were stimulated using an STG4002-1.6mA stimulus generator (Multichannel Systems) controlled by MC_Stimulus software. We used 50-μs, 0.8-mA square-wave pulses to evoke CAPs, recorded by a USB-ME16-FAI system (Multichannel Systems) with a 4× gain preamplifier. Data were collected at 50 kHz by MC_Rack software and analyzed using a custom MATLAB script available on GitHub (https://github.com/EIN-lab/CAP-analysis). CAP responses, characterized by three peaks indicating different axonal conduction speeds^10,28,108^, decrease peak amplitude and increase latency during high-frequency stimulation^28^. The analysis focused on the second CAP peak, measuring latency from stimulus onset to peak. The pCAP area was used to integrate the amplitude and latency changes of the first two peaks, reflective of large and medium-sized axons. Stimulation protocols involved an initial 0.4-Hz stimulation for 1 min for baseline values, followed by 30 s or 1 min at 10, 25 or 50 Hz, then 4–5 min recovery at 0.4 Hz. High-frequency CAPs were recorded every second. GD and MI experiments used a 0.1-Hz stimulation protocol. Nerve excitability was assessed by varying stimulus intensity from 0.1 to 1 mA, measuring the AUC of graded CAPs, with the CAP area expressed as a percentage of the maximum CAP area at 1 mA.

### Calcium imaging and analysis

GCaMP6s imaging involved exciting the sensor at 940 nm with laser powers between 5 and 10 mW. Fluorescence emissions were captured using a GaAsP photomultiplier tube (Hamamatsu Photonics) and a 520/70-nm band-pass filter (Semrock). Overview images were taken at 512 × 512 resolution with a 3.2-μs pixel dwell time. For Ca^2+^ imaging in OLs or axons, the selected field of view, 15–20 μm below the nerve surface, used 7–8× digital zoom, capturing images at 2.96 Hz and 128 × 128 resolution with a 12.8-μs pixel dwell time. Data analysis was conducted using MATLAB (MathWorks, R2015b) with the CHIPS toolbox^109^ (https://github.com/EIN-lab/CHIPS), as previously described^78,84,110^. Regions of interest (ROIs) around OL somas were manually outlined in ImageJ, with ROI masks inputted into the CHIPS pipeline. For axons, whole frames were analyzed. Image sequences underwent motion correction, noise reduction using a two-dimensional spatial Gaussian filter (σxy = 2 µm) and a temporal moving-average filter (width = 1 s), and background noise (defined as the bottom first-percentile pixel value) subtraction. Signal vectors (Δ*F*/*F*) from each ROI or frame were computed using ten frames before stimulation onset as the baseline. Ca^2+^ responses were quantified by the AUC during a 30-s stimulation period. Pharmacological effects were assessed through paired analysis comparing the responses before and after drug application, averaged over two to three sessions to minimize variability. The numbers of mice and cells are detailed in the figure legends.

### Metabolite imaging and analysis

The FRET-based metabolite sensors (Laconic, ATeam1.03, FLIIP) were excited at 870 nm using laser powers of 5–15 mW. Donor and acceptor fluorescence signals were simultaneously collected using two photomultiplier tubes, a 560-nm edge dichroic beam splitter (BrightLine, Semrock), and band-pass filters of 545/55 nm (yellow channel) and 475/50 nm (blue channel). Images were captured at 256 × 256 resolution with a 6.4-μs pixel dwell time, every 2 or 10 s. FRET analysis used a custom MATLAB script, available at https://gitlab.com/einlabzurich/fretanalysis. Image time series were smoothed using a five-image moving average, with the number of images determined after testing various time windows to preserve temporal dynamics. Background removal was achieved by thresholding, with average whole-frame intensities extracted for each channel. Donor-to-acceptor (Laconic) or acceptor-to-donor (ATeam1.03 and FLIIP) ratios were calculated and normalized to baselines or quasi-zero time points. For visualizing OL or axonal structures while maintaining quantitative FRET ratio data, a color scale was applied to ratio images obtained by pixel-by-pixel division of two channels over an average of 20 frames. RGB images were converted to YCbCr color space, where Cb and Cr represent color and Y represents brightness. The *y* coordinate was replaced with the square root of the sum of donor and acceptor images, and the image was reconverted to RGB.

### Immunohistochemistry

Mice were anesthetized with pentobarbital and perfused with 2% paraformaldehyde (PFA) in PBS. Optic nerves were postfixed for 1 h in 4% PFA, embedded in frozen section medium (Thermo Fisher Scientific) and cut into 12-μm longitudinal sections using a Leica CM3050 S cryostat. Sections were placed on SuperFrost Plus slides (Thermo Fisher Scientific). For immunohistochemistry, slides were first treated with 0.3% Triton X-100 in 50 mM Tris buffer (pH 7.4) with 4% normal donkey serum for 1 h at room temperature. Primary antibodies (Table 1) were incubated overnight at 4 °C in the same solution. Secondary antibodies, mixed with 0.05% Tris–Triton and 4% serum, were applied for 1 h at room temperature. DAPI was added for nuclear staining. Confocal images and z stacks (10 µm) were acquired with a Zeiss LSM 700 or Zeiss LSM 800 confocal laser scanning microscope equipped with a 40× objective (Plan-Apochromat, numerical aperture 1.4, Oil DIC (UV) VIS–IR). Image analysis was performed with ImageJ (Fiji version 1.52p). For GFAP and IBA1 analysis, maximum intensity projections were binarized and the fluorescent particle area was determined. For quantifications, two images per section and four sections per animal were analyzed.

**Table 1:** Antibody information

Antibody	Host species, type	Method, dilution	Source, cat. no.
Anti-CC1	Mouse, monoclonal	IHC, 1:100	Calbiochem, cat. no. OP80 (clone 5.24)
Anti-GFP	Chicken, polyclonal	IHC, 1:1,000	Aves Labs, cat. no. GFP-1020
Anti-GFAP	Chicken, polyclonal	IHC, 1:2,000	Abcam, cat. no. ab4674
Anti-IBA1	Rabbit, polyclonal	IHC, 1:1,000	FUJIFILM Wako Chemicals, cat. no. 019-19741
Anti-Kir4.1	Rabbit, polyclonal	IB, 1:1,000	Alomone, cat. no. APC-035
Anti-MCT1/SLC16A1	Rabbit, polyclonal	IB, 1:500	Produced by Kathrin Kusch^116^
Anti-GLUT1	Rabbit, polyclonal	IB, 1:500	Produced by Kathrin Kusch^117^
Anti-CNP	Mouse, monoclonal	IB, 1:1,000	Sigma, cat. no. C 5922 (clone 11-5B)
Anti-PLP	Rabbit, polyclonal	IB, 1:5,000	A431 (ref. ^118^)
Anti-MOG	Mouse, monoclonal	IB, 1:5,000	Creative Biolabs, cat. no. PABZ-152 (clone 8-18C5)
Anti-ATP1α1	Mouse, monoclonal	IB, 1:1,000	Abcam, cat. no. ab7671 (clone 464.6)
Anti-ATP1α3	Mouse, monoclonal	IB, 1:1,000	Abcam, cat. no. ab2826 (clone XVIF9- G10)
Anti-mouse IgG HRP	Goat, polyclonal	IB, 1:10,000	Jackson ImmunoResearch, cat. no. 115-035-003
Anti-rabbit IgG HRP	Goat, polyclonal	IB, 1:10,000	Jackson ImmunoResearch, cat. no. 111-035-003
Anti-mouse Cy3	Donkey, polyclonal	IHC, 1:700	Jackson ImmunoResearch, cat. no. 715-165-151
Anti-rabbit Cy3	Donkey, polyclonal	IHC, 1:700	Jackson ImmunoResearch, cat. no. 711-165-152
Anti-chicken Alexa 488	Donkey, polyclonal	IHC, 1:700	Jackson ImmunoResearch, cat. no. 711-545-152

IgG, immunoglobulin G; HRP, horseradish peroxidase; IHC, immunohistochemistry; IB, immunoblot.

### EM and analysis

Mice were anesthetized using isoflurane before decapitation and optic nerve extraction. Optic nerves were immediately transferred to a fixative solution (4% PFA, 2.5% glutaraldehyde in phosphate buffer with 0.5% NaCl, pH 7.4) and fixed overnight at 4 °C. Tissue preparation and EM were carried out as previously described^111^. Briefly, following postfixation with 2% OsO_4_ (Science Services) in 0.1 M phosphate buffer (pH 7.3) and acetone dehydration, tissue fragments were embedded in EPON (Serva). Ultrathin sections were cut with a Leica UC7 ultramicrotome (Leica) and then stained using UranyLess (Science Services). EM pictures were captured with a Zeiss EM912 electron microscope (Zeiss) equipped with an on-axis 2k charge-coupled device camera (TRS). EM image analysis was performed using ImageJ (Fiji, version 1.52p). Axonal diameters and *g* ratios (ratio of axon diameter to the diameter including the myelin sheath) were analyzed using five random overview EM pictures (at 8,000× magnification), with 250 axons evaluated per animal. Calculations were based on circular areas equivalent to the measured areas. For axonal pathology assessment, eight to ten EM images per animal were analyzed. The experimenters were blinded to the genotypes.

### Proteomics and analysis

Optic nerves from 2.5-month-old Kir4.1 cKO mice and littermate controls were extracted following deep isoflurane anesthesia. For tissue homogenization, we used PreOmics lysis buffer with 0.5-mm zirconium oxide beads (ZROB05, Next Advance) in a Bullet Blender (BBX24, Next Advance), with two 15-s cycles set at speed 10. TMT-based quantitative proteomics analysis was performed at the Functional Genomics Center Zurich. Protein concentrations were measured using the Lunatic UV–Vis spectrophotometer (Unchained Labs). Processing involved the iST kit (PreOmics) and encompassed treatment with ‘Lyse’ buffer, boiling, digestion and centrifugation. Peptides captured by the iST filter were washed, eluted, dried and labeled with TMT10plex reagent (Thermo Fisher Scientific, 90110) in acetonitrile (Sigma-Aldrich) and 50 mM TEAB (pH 8.5), followed by quenching. Equal TMT channel amounts formed the combined sample, prefractionated using high-pH reverse-phase chromatography on an XBridge Peptide BEH C18 column (Waters). For MS, samples were analyzed on an Orbitrap Fusion Lumos (Thermo Fisher Scientific), with data-dependent acquisition settings optimized for MS spectra and MS/MS recordings in the Orbitrap. Proteomics data were handled using the local laboratory information management system^112^. Proteome Discoverer (version 2.4) processed the raw MS data, with protein identification through the Sequest HT engine against the *Mus musculus* reference proteome (UniProt, 20190709). Carbamidomethylation and TMT (+229.163 Da, peptide N-terminus and K) were set as fixed modifications, whereas methionine oxidation and N-terminal protein acetylation were set as variable modifications. Enzyme specificity was set to trypsin, allowing a minimal peptide length of six amino acids and a maximum of two missed cleavages. The maximum FDR for peptides was set to 0.01. Protein fold changes were computed based on intensity values. A set of functions implemented in R package prolfqua^113^ was used to filter for proteins with two or more peptides. Data normalization used a modified robust *z*-score transformation, preserving the original data variance. For differential analysis, we fitted the linear model to every protein, computed contrasts, and moderated the variance, *t* statistics and *P* values^114^. FDRs were determined from *P* values using Benjamini–Hochberg adjustment. GSEA was conducted using WebGestalt.org.

### Myelin purification and immunoblotting

A myelin-enriched lightweight membrane fraction was purified from the brains of 2.5-month-old Kir4.1 cKO mice and controls, using sucrose density centrifugation and osmotic shocks as previously described^115^. Brain tissue was extracted after decapitation following deep anesthesia using isoflurane. Protein concentrations in brain lysates and myelin fractions were determined using the DC protein assay kit (Bio-Rad) following the manufacturer’s instructions and measured using the Eon high-performance microplate spectrophotometer (BioTek). Immunoblotting was carried out as previously outlined^18^. Myelin fraction samples were diluted in 4× SDS sample buffer (glycerol 40% (w/v), Tris–HCl (pH 6.8) 240 mM, SDS 8% (w/v), bromophenol blue 0.04% (w/v)), with 5% dithiothreitol added as a reducing agent. Before use, samples were heated at 40 °C for 10 min. For protein separation by SDS–PAGE, the Mini-PROTEAN Handcast system (Bio-Rad) was used with self-casted acrylamide gels (10–15%). Samples were loaded at 5–15 µg per well (depending on the protein of interest) next to 5 µl of prestained protein ladder (PageRuler, Thermo Fisher Scientific). Proteins were separated by constant current (200 V) for 45–60 min. Immunoblotting was carried out with a Novex semi-dry blotter (Invitrogen), and proteins were transferred to an activated (100% ethanol, 1 min; followed by two washing steps with water) polyvinylidene fluoride membrane (Roche Diagnostics, cat. no. 03010040001) at 20 V for 40 min. After blotting, membranes were blocked in 1× TBS containing 5% nonfat dry milk (Frema) and 0.05% Tween-20 for 1 h at room temperature. Primary antibodies were diluted in 5 ml of blocking buffer and incubated overnight at 4 °C with horizontal rotation. Membranes were washed three times with TBS-T for 5–10 min each and incubated for 1 h with secondary HRP antibodies diluted in a blocking buffer. Membranes were washed three times with TBS-T for 5–10 min. Detection was carried out using enhanced chemiluminescent detection (ECL) according to the manufacturer’s instructions (Western Lightning Plus-ECL or SUperSignal West Femto Maximum Sensitivity Substrate, Thermo Fisher Scientific). Immunoblots were scanned using the ECL imager Chemostar (Intas Science Imaging). Normalization was performed with Fast Green total protein stain. For antibody information, see Table 1.

### Real-time qPCR

Mice were deeply anesthetized using isoflurane and then decapitated. Next, optic nerves were quickly dissected. The tissues were homogenized in 350 µl RNeasy lysis buffer (RLT, Qiagen) with 0.5-mm zirconium oxide beads (ZROB05, Next Advance) in a Bullet Blender tissue homogenizer (BBX24, Next Advance; two cycles of 15 s on speed 10). For RNA extraction and cDNA synthesis, the RNeasy Micro kit (Qiagen) and SensiFAST cDNA synthesis kit (Bioline) were used according to the manufacturer’s instructions. Real-time qPCR was performed using EvaGreen (HOT FIREPol, Solis BioDyne) and a 7900HT Fast Real-Time PCR System (Applied Biosystems, software SDS 2.4). For mRNA expression analysis, the following primers were used: for Kir4.1 (*Kcnj10*) (5′-TCT GTT CAT CTG TCC CGC TGC-3′, 5′-GAC GTC ATC TTG GCT CGA AGG-3′), for MCT1 (*Slc16a1*) (5′-ATT GTG GAA TGC TGC CCT GT-3′, 5′-TAC CCG CGA TGA TGA GGA TC-3′), for GLUT1 (*Slc2a1*) (5′-ATC TTC GAG AAG GCA GGT GTG-3′, 5′-CGC TCT ACA ACA AAC AGC GAC-3′) and for GLUT3 (*Slc2a3*) (5′-GTG ACT GTG CTG GAG CTC TT-3′, 5′-CCG CGT CCT TGA AGA TTC CT-3′). *Actb* served as an endogenous gene control (5′-CTT CCT CCC TGG AGA AGA GC-3′, 5′-ATG CCA CAG GAT TCC ATA CC-3′). Three technical replicates were averaged for each reaction for each animal.

### Statistical analyses

Intergroup comparisons were performed by a two-sided paired *t* test or Student’s *t* test, as specified in the figure legends. For analyses involving multiple comparisons, data were subjected to either one-way or two-way ANOVA. All statistical analyses were conducted using GraphPad Prism 9 or R (v.3.2.2, R Core Team, 2015). Datasets had to pass the Shapiro–Wilk test for normality before being subjected to Student’s *t* test and ANOVA or undergo nonparametric tests to determine statistical significance. Equal variances were assumed but not tested. Significance levels were defined as follows: *P < 0.05, **P < 0.01, ***P < 0.001. Data are presented as individual values with mean ± s.e.m. or as box-and-whisker plots. In the box-and-whisker plots, the ‘+’ symbol represents the mean; the center line indicates the median; the box extends from the 25th to 75th percentile; and the whiskers extend from the 5th to 95th percentile. *P* values and sample sizes are stated in the figure legends. Sample sizes were not determined in advance as they were constrained by the availability of age-matched transgenic mouse cohorts. Where applicable, experimenters were blinded to the genotype.

### Reporting summary

Further information on research design is available in the Nature Portfolio Reporting Summary linked to this article.

## Online content

Any methods, additional references, Nature Portfolio reporting summaries, source data, extended data, supplementary information, acknowledgements, peer review information; details of author contributions and competing interests; and statements of data and code availability are available at 10.1038/s41593-023-01558-3.

### Supplementary information

Reporting Summary

Supplementary Video 1 Example time-lapse recording of GCaMP6s-expressing OLs responding to a 30-s, 50-Hz optic nerve stimulation. Images were acquired at 2.96 Hz, and the video plays at 160 fps. The stimulation period is indicated by the white square (top right corner). Scale bar, 10 µm.

Supplementary Video 2 Example time-lapse recording of GCaMP6s-expressing optic nerve axons responding to a 30-s, 50-Hz optic nerve stimulation. Images were acquired at 2.96 Hz, and the video plays at 160 fps. The stimulation period is indicated by the white square (top right corner). Scale bar, 10 µm.

Supplementary Data 1 Raw and normalized MS proteomics data reported in this study.

Supplementary Data 2 Uncropped western blots for Fig. 4e.

## Data Availability

Mass spectrometry proteomics data reported in this study (see also Supplementary Data 1, containing raw and normalized proteomics data) are deposited at the ProteomeXchange PRIDE with dataset identifier PXD046207. Further data and resources are available upon request from the corresponding author.

## Code availability

MATLAB code for calcium analysis^109^ is available at https://github.com/EIN-lab/CHIPS; code for CAP analysis^28^ is available at https://github.com/EIN-lab/CAP-analysis; and code for FRET image analysis is available at https://gitlab.com/einlabzurich/fretanalysis.
