# Supplementary material for: Oligodendrocyte–axon metabolic coupling is mediated by extracellular K+ and maintains axonal health
Source: Nat Neurosci. 2024 Jan 24;27(3):433–48. doi: 10.1038/s41593-023-01558-3 (PMC10917689; doi:10.1038/s41593-023-01558-3)
Supplement: Supplementary file 1 — Reporting Summary [file 41593_2023_1558_MOESM1_ESM.pdf]

nature portfolio

Corresponding author(s): Aiman S. Saab

Last updated by author(s): Nov 20, 2023

Reporting Summary

Nature Portfolio wishes to improve the reproducibility of the work that we publish. This form provides structure for consistency and transparency in reporting. For further information on Nature Portfolio policies, see our Editorial Policies and the Editorial Policy Checklist.

Statistics

For all statistical analyses, confirm that the following items are present in the figure legend, table legend, main text, or Methods section.

n/a	Confirmed	
☐	☒	The exact sample size (*n*) for each experimental group/condition, given as a discrete number and unit of measurement
☐	☒	A statement on whether measurements were taken from distinct samples or whether the same sample was measured repeatedly
☐	☒	The statistical test(s) used AND whether they are one- or two-sided<br>*Only common tests should be described solely by name; describe more complex techniques in the Methods section.*
☐	☒	A description of all covariates tested
☒	☐	A description of any assumptions or corrections, such as tests of normality and adjustment for multiple comparisons
☐	☒	A full description of the statistical parameters including central tendency (e.g. means) or other basic estimates (e.g. regression coefficient) AND variation (e.g. standard deviation) or associated estimates of uncertainty (e.g. confidence intervals)
☐	☒	For null hypothesis testing, the test statistic (e.g. *F*, *t*, *r*) with confidence intervals, effect sizes, degrees of freedom and *P* value noted<br>*Give P values as exact values whenever suitable.*
☒	☐	For Bayesian analysis, information on the choice of priors and Markov chain Monte Carlo settings
☒	☐	For hierarchical and complex designs, identification of the appropriate level for tests and full reporting of outcomes
☒	☐	Estimates of effect sizes (e.g. Cohen's *d*, Pearson's *r*), indicating how they were calculated

Our web collection on statistics for biologists contains articles on many of the points above.

Software and code

Policy information about availability of computer code

Data collection	CAP recordings: perfusion chamber (Haas Top, Harvard Apparatus); USB-ME16-FAI acquisition system (Multichannel Systems) connected to a USB-ME16-FAI preamplifier (gain 4x; Multichannel Systems) and data collected with the acquisition software MC_Rack (Multichannel Systems)<br>Two-photon imaging: custom-built two-photon microscope (Mayrhofer et al., 2015), equipped with a tunable pulsed Ti:Sapphire laser (Chameleon Ultra II; Coherent) and a 25x water immersion objective (XLPLN 25x/1.05 WMP2, Olympus). The microscope was controlled by a customized version of ScanImage (r3.8.1; Janelia Research Campus, (Pologruto et al., 2003)). Fluorescence emission was detected with a GaAsP photomultiplier tube (PMT; Hamamatsu Photonics) using band-pass filter 520/70 nm (Semrock) or a dichroic beam-splitter (560 nm edge, BrightLine; Semrock) and two band-pass filters 545/55 nm and 475/50 nm (Semrock).<br>Immunohistochemistry: Confocal images were acquired with a Zeiss LSM 700 or Zeiss LSM 800 confocal laser scanning microscope equipped with a 40× objective (Plan-Apochromat, NA 1.4, Oil DIC (UV) VIS-IR).<br>Electron microscopy (EM): EM pictures were captured with a Zeiss EM912 electron microscope (Carl Zeiss Microscopy GmbH, Oberkochen, Germany) equipped with an on-axis 2k CCD camera (TRS, Moorenweis, Germany).<br>Proteomics: For mass spectrometry analysis, an Orbitrap Fusion Lumos (Thermo Scientific) was used, which was equipped with a Digital PicoView source (New Objective) and coupled to a M-Class UPLC (Waters).<br>Immunoblotting: Detection was carried out using enhanced chemiluminescent detection (ECL) according to the manufacturer's instructions (Western Lightning Plus-ECL or SUperSignal West Femto Maximum Sensitive Substrate; Thermo Fisher Scientific, St Leon-Rot, Germany). Immunoblots were scanned using ECL Chemostar (Intas Science Imaging, Göttingen, Germany).
Data analysis	MATLAB (MathWorks, R2015b, R2019a), ImageJ (Fiji version 1.52p), GraphPad Prism 9, R (v.3.2.2, R Core Team, 2015); CAP recordings were analyzed using a custom-written MATLAB script available at GitHub (https://github.com/EIN-lab/CAPanalysis); Image analysis using custom toolbox CHIPS (Barrett et al., 2018) available on GITHUB (https://github.com/EIN-lab/CHIPS). Code used for FRET image analysis is available at GitHub (https://gitlab.com/einlabzurich/fretanalysis). Functions implemented in the R package prolfqua were used for proteomics data

processing.

For manuscripts utilizing custom algorithms or software that are central to the research but not yet described in published literature, software must be made available to editors and reviewers. We strongly encourage code deposition in a community repository (e.g. GitHub). See the Nature Portfolio guidelines for submitting code & software for further information.

Data

Policy information about availability of data

All manuscripts must include a data availability statement. This statement should provide the following information, where applicable:

- Accession codes, unique identifiers, or web links for publicly available datasets
- A description of any restrictions on data availability
- For clinical datasets or third party data, please ensure that the statement adheres to our policy

Mass spectrometry proteomics data reported in this study (see also Table S1 containing raw and normalized proteomics data) are deposited at the ProteomeXchange PRIDE with the dataset identifier PXD046207. The mus musculus reference proteome was downloaded from UniProt, 20190709. GSEA was carried out with the WEB-based GEne SeT AnaLysis Toolkit (WebGestalt.org). Further data and resources are available upon reasonable request from corresponding author.

Research involving human participants, their data, or biological material

Policy information about studies with human participants or human data. See also policy information about sex, gender (identity/presentation), and sexual orientation and race, ethnicity and racism.

Reporting on sex and gender	N/A
Reporting on race, ethnicity, or other socially relevant groupings	N/A
Population characteristics	N/A
Recruitment	N/A
Ethics oversight	N/A

Note that full information on the approval of the study protocol must also be provided in the manuscript.

Field-specific reporting

Please select the one below that is the best fit for your research. If you are not sure, read the appropriate sections before making your selection.

☒ Life sciences ☐ Behavioural & social sciences ☐ Ecological, evolutionary & environmental sciences

For a reference copy of the document with all sections, see nature.com/documents/nr-reporting-summary-flat.pdf

Life sciences study design

All studies must disclose on these points even when the disclosure is negative.

Sample size	The provision of age-matched transgenic mouse cohorts limited the ability to determine sample sizes in advance. No statistical methods were used to predetermine sample size. Sample sizes used in this study are equivalent to the standard in the field.
Data exclusions	ometimes sensor expression was too weak (e.g. due to insufficient tamoxifen-induced Cre-mediated expression or AAV delivery), these animals were excluded from experiments.
Replication	Number of repetitions (individual data points from each cells and/or animal) are indicated in figures or figure legends.
Randomization	Selection of mice was based on genotype or wildtype mice were ordered from Charles River. Mice were randomly assigned to experimental groups, depending on availability of transgenic cohorts.
Blinding	Overall, during data collection experimenters were not blinded to the conditions. However, experimenter who collected EM dataset was blinded to the experimental conditions. Whenever possible, investigators were blinded for data analysis, especially for EM image analysis.

Reporting for specific materials, systems and methods

We require information from authors about some types of materials, experimental systems and methods used in many studies. Here, indicate whether each material, system or method listed is relevant to your study. If you are not sure if a list item applies to your research, read the appropriate section before selecting a response.

Materials & experimental systems

n/a	Involved in the study
☐	☒ Antibodies
☒	☐ Eukaryotic cell lines
☒	☐ Palaeontology and archaeology
☐	☒ Animals and other organisms
☒	☐ Clinical data
☒	☐ Dual use research of concern
☒	☐ Plants

Methods

n/a	Involved in the study
☒	☐ ChIP-seq
☒	☐ Flow cytometry
☒	☐ MRI-based neuroimaging

Antibodies

Antibodies used

Antibody information. IHC, immunohistochemistry, IB, immunoblot

α-CC1 mouse, monoclonal (clone 5.24) IHC, 1:100 Calbiochem, Cat# OP80
α-GFP chicken, polyclonal IHC, 1:1000 Aves Labs, Cat# GFP-1020
α-GFAP chicken, polyclonal IHC, 1:2000 Abcam, Cat# ab4674
α-IBA1 rabbit, polyclonal IHC, 1:1000 FUJIFILM Wako Chemicals, Cat# 019-19741
α-Kir4.1 rabbit, polyclonal IB, 1:1000 Alomone, Cat# APC-035
α-MCT1 / SLC16A1 rabbit, polyclonal IB, 1:500 prod. by Kathrin Kusch (Stumpf et al., 2019): antibody produced by Kathrin Kusch from the Department of Neurogenetics, Max Planck Institute for Multidisciplinary Sciences, Göttingen, Germany.
α-GLUT1 rabbit, polyclonal IB, 1:500 prod. by Kathrin Kusch (Berghoff et al., 2017): antibody produced by Kathrin Kusch from the Department of Neurogenetics, Max Planck Institute for Multidisciplinary Sciences, Göttingen, Germany.
α-CNP mouse, monoclonal (clone 11-5B) IB, 1:1000 Sigma, Cat# C 5922
α-PLP rabbit, polyclonal IB, 1:5000 A431; (Jung et al., 1996): provided by Hauke Werner from the Department of Neurogenetics, Max Planck Institute for Multidisciplinary Sciences, Göttingen, Germany.
α-MOG (clone 8-18C5) mouse, monoclonal IB, 1:5000 (Linnington et al. 1984), Creative Biolabs, Cat# PABZ-152
α-ATP2a1 mouse, monoclonal (clone 464.6) IB, 1:1000 Abcam, Cat# ab7671
α-ATP1a3 mouse, monoclonal (clone XVIF9-G10) IB, 1:1000 Abcam, Cat# ab2826
α -mouse IgG HRP goat, polyclonal IB, 1:10000 Jackson ImmunoResearch, Cat# 115-035-003
α -rabbit IgG HRP goat, polyclonal IB, 1:10000 Jackson ImmunoResearch, Cat# 111-035-003
α-mouse Cy3 donkey IHC, 1:700 Jackson ImmunoResearch, Cat# 715-165-151
α-rabbit Cy3 donkey IHC, 1:700 Jackson ImmunoResearch, Cat# 711-165-152
α-chicken Alexa 488 donkey IHC, 1:700 Jackson ImmunoResearch, Cat# 711-545-152

Validation

Quality control information and relevant citations are available at manufacturer's website.
For α-CC1: https://www.merckmillipore.com/CH/de/product/Anti-APC-Ab-7-Mouse-mAb-CC-1,EMD_BIO-OP80?ReferrerURL=https%3A%2F%2Fwww.google.com%2F; For α-GFP: https://www.aveslabs.com/products/anti-green-fluorescent-protein-antibody-gfp; for α-GFAP: https://www.abcam.com/products/primary-antibodies/gfap-antibody-ab4674.html; for α-IBA1: https://labchem-wako.fujifilm.com/us/product/detail/W01W0101-1974.html; for α-Kir4.1: https://www.alomone.com/p/anti-kir4-1/APC-035; for α-CNP: https://www.sigmaaldrich.com/CH/de/product/sigma/c5922; for α-MOG: https://www.creativebiolabs.net/Anti-MOG-Recombinant-Antibody-clone-8-18C5-24503.htm; for α-ATP2a1: https://www.abcam.com/products/primary-antibodies/alpha-1-sodium-potassium-atpase-antibody-4646-ab7671.html; for α-ATP1a3: https://www.abcam.com/products/primary-antibodies/atp1a3-antibody-xvif9-g10-ab2826.html; for α -mouse IgG HRP: https://www.jacksonimmuno.com/catalog/products/115-035-003; for α -rabbit IgG HRP: https://www.jacksonimmuno.com/catalog/products/111-035-003; for α-mouse Cy3 donkey: https://www.jacksonimmuno.com/catalog/products/715-165-151; for α-rabbit Cy3 donkey: https://www.jacksonimmuno.com/catalog/products/711-165-152; for α-chicken Alexa 488 donkey: https://www.jacksonimmuno.com/catalog/products/711-545-152. Antibody validation for use in immunoblotting were confirmed by cited publications for α-MCT1 / SLC16A1 (Stumpf et al., 2019), for α-GLUT1 (Berghoff et al., 2017) and for α-PLP (Jung et al., 1996).

Animals and other research organisms

Policy information about studies involving animals; ARRIVE guidelines recommended for reporting animal research, and Sex and Gender in Research

Laboratory animals

PLP-CreERT;RCL-GCaMP6s mice were generated by crossing PLP-CreERT mice (RRID:IMSR_JAX:005975) (Doerflinger et al., 2003) with ROSA26-floxed-STOP-GCaMP6s mice (Ai96; RRID:IMSR_JAX:024106) (Madisen et al., 2015). Age 6 to 20 weeks.
Kir4.1fl/fl;MOGiCre mice (Larson et al., 2018) were obtained from crosses of mice carrying the floxed Kcnj10 (Kir4.1fl/fl) (Djukic et al., 2007) allele with MOGi-Cre mice (Hövelmeyer et al., 2005). Age 6 weeks to 8 months.
For experiments in wildtype mice Charles River C57BL/6 mice were used. Age 8 weeks to 5 months.

Wild animals

no wild animals were used in the study

Reporting on sex

Both male and female mice were used for experiments

Field-collected samples	no field collected samples were used in the study
Ethics oversight	All animal experiments were permitted by the local veterinary authorities in Zurich, in agreement with the guidelines of Swiss Animal Protection Law, Veterinary Office, Canton Zurich (Animal Welfare Act of 16 December 2005 and Animal Welfare Ordinance of 23 April 2008).

Note that full information on the approval of the study protocol must also be provided in the manuscript.
