# Supplementary material for: Oligodendrocyte–axon metabolic coupling is mediated by extracellular K+ and maintains axonal health
Source: Nat Neurosci. 2024 Jan 24;27(3):433–48. doi: 10.1038/s41593-023-01558-3 (PMC10917689; doi:10.1038/s41593-023-01558-3)
Supplement: Supplementary file 5 — Uncropped western blots for Fig. 4e. [file 41593_2023_1558_MOESM5_ESM.pdf]

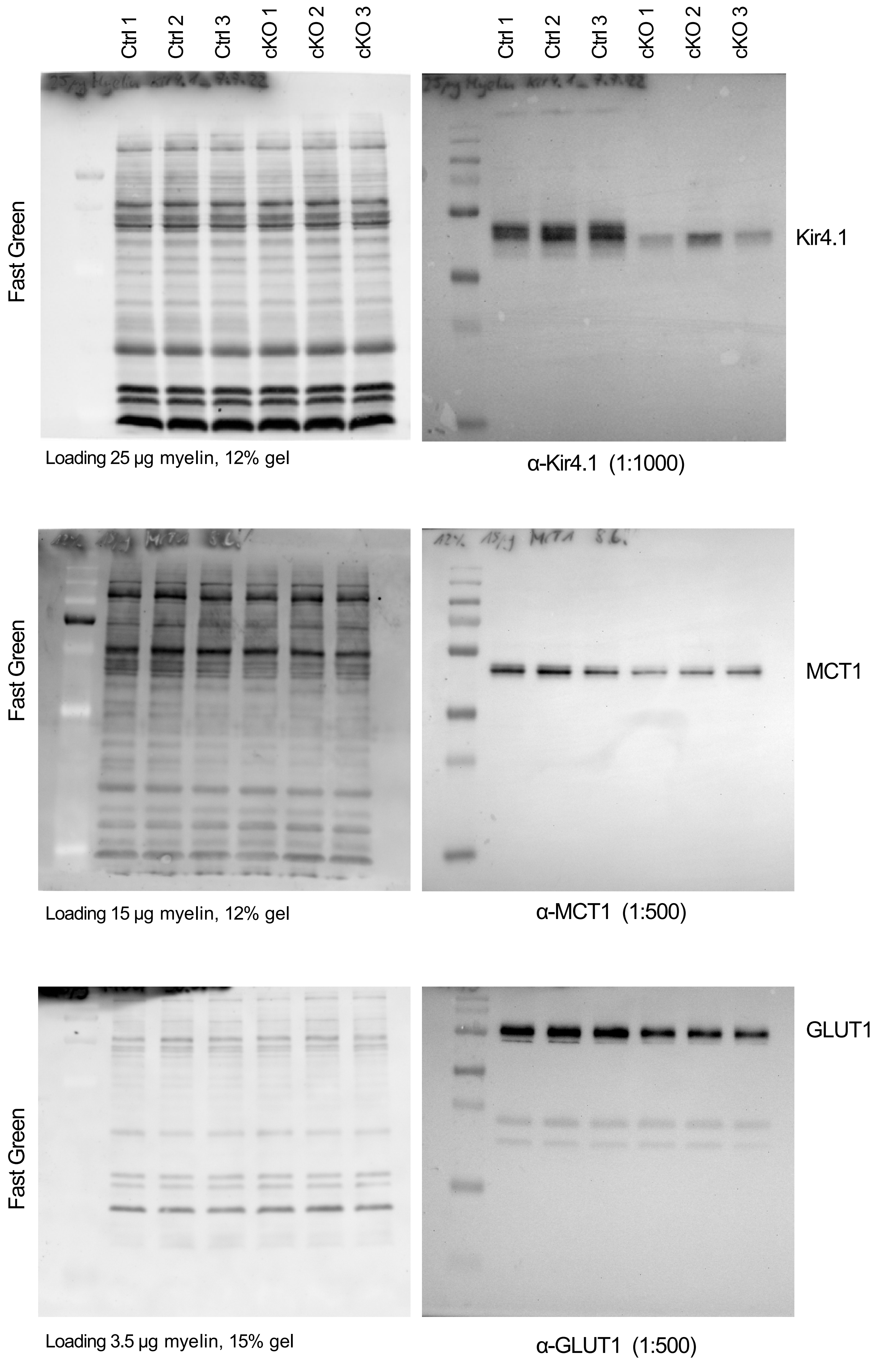

Ctrl 1
Ctrl 2
Ctrl 3
cKO 1
cKO 2
cKO 3
Fast Green
Kir4.1
Loading 25 µg myelin, 12% gel
α-Kir4.1 (1:1000)
Fast Green
MCT1
Loading 15 µg myelin, 12% gel
α-MCT1 (1:500)
Fast Green
GLUT1
Loading 3.5 µg myelin, 15% gel
α-GLUT1 (1:500)

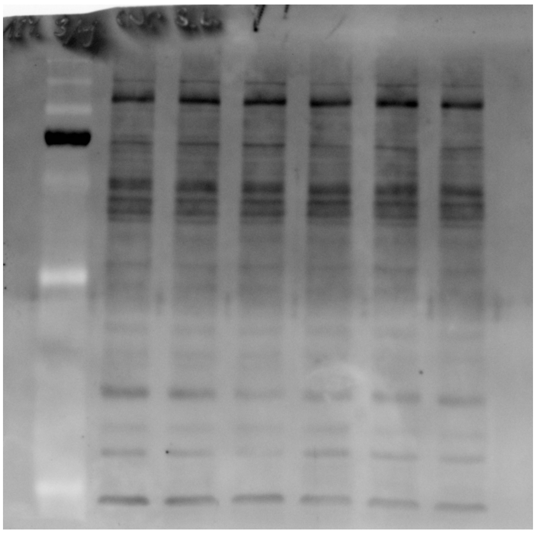

Loading 3 µg myelin, 12% gel

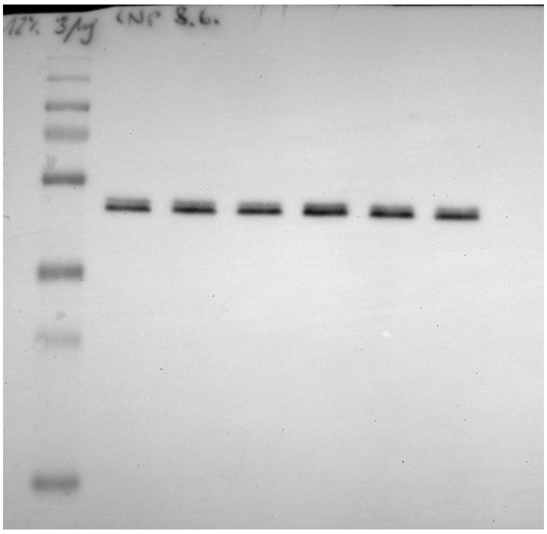

α-CNP (1:1000)

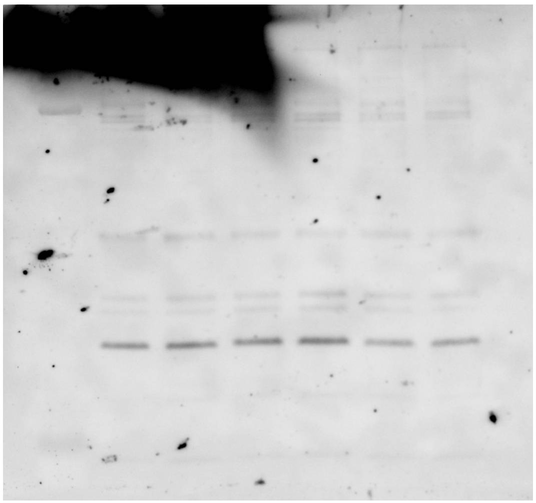

Loading 1 µg myelin, 15% gel

membrane was cut horizontally

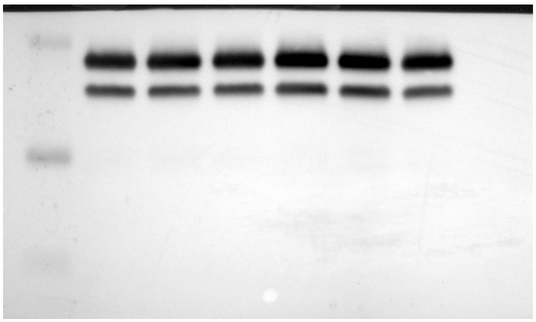

α-PLP (1:5000)

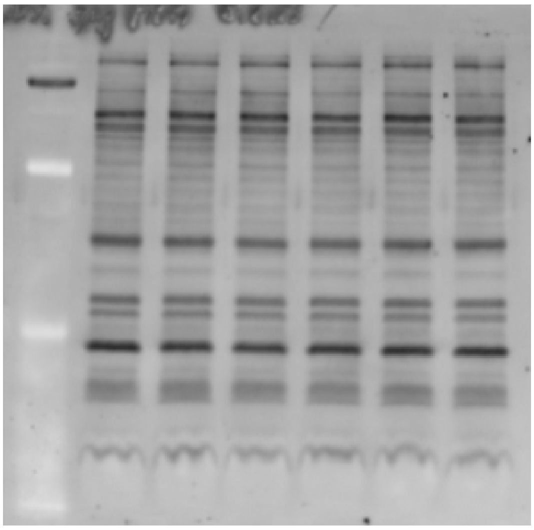

Loading 5 µg myelin, 15% gel

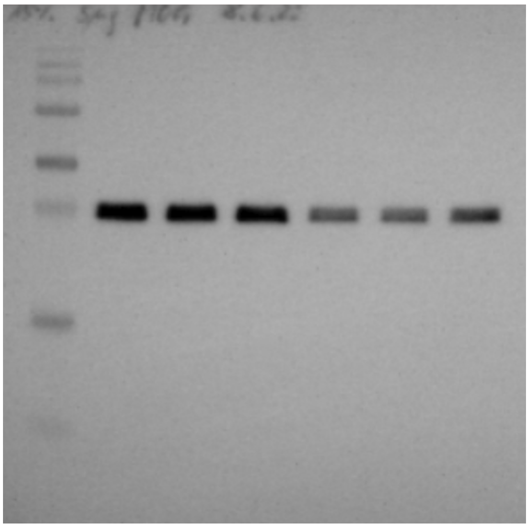

α-MOG (1:5000)

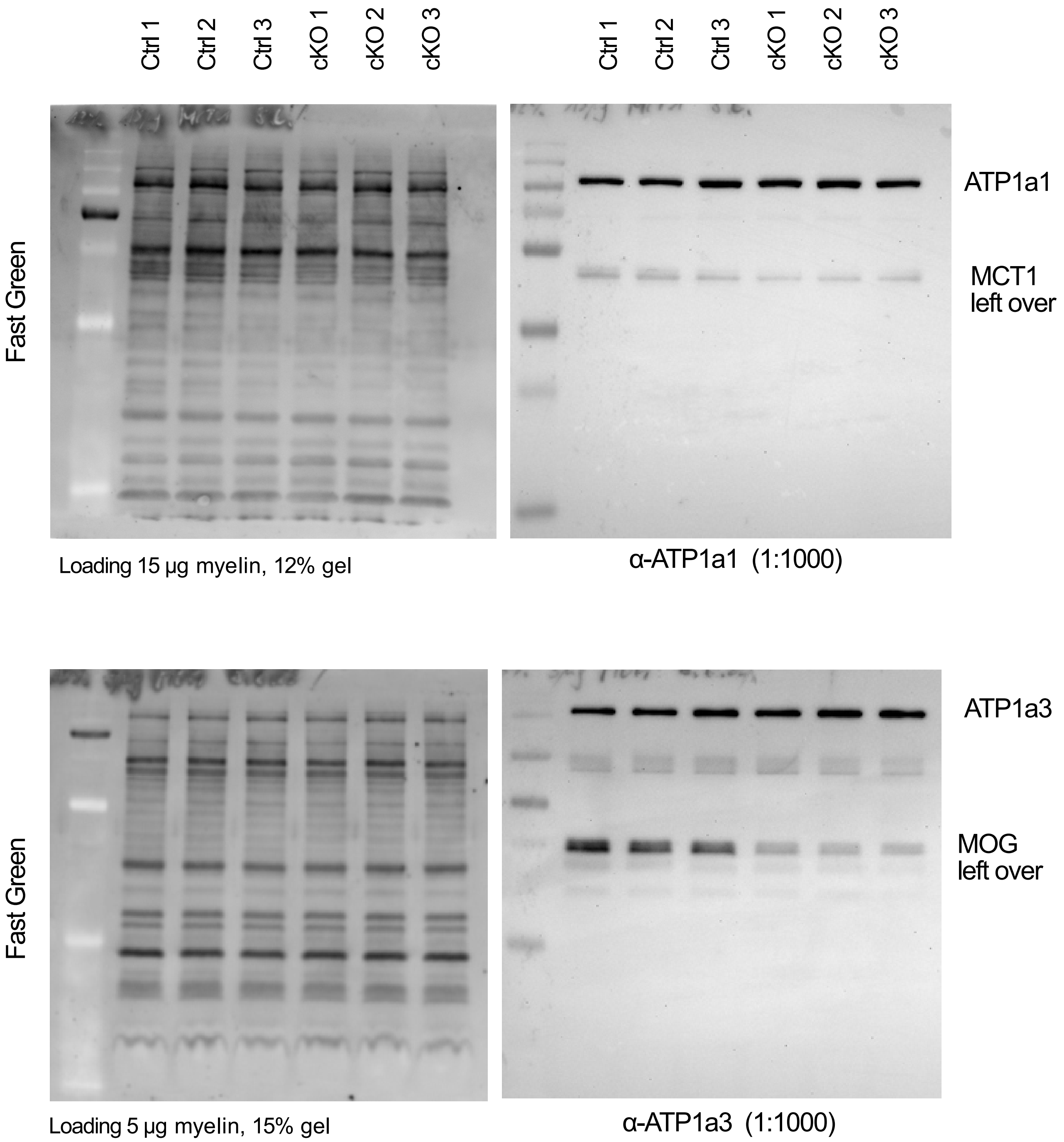
Ctrl 1
Ctrl 2
Ctrl 3
cKO 1
cKO 2
cKO 3
Fast Green
Loading 15 µg myelin, 12% gel
ATP1a1
MCT1
left over
α-ATP1a1 (1:1000)
Fast Green
Loading 5 µg myelin, 15% gel
ATP1a3
MOG
left over
α-ATP1a3 (1:1000)
